# Engineering *Shewanella oneidensis*‐Carbon Felt Biohybrid Electrode Decorated with Bacterial Cellulose Aerogel‐Electropolymerized Anthraquinone to Boost Energy and Chemicals Production

**DOI:** 10.1002/advs.202407599

**Published:** 2024-08-19

**Authors:** Qijing Liu, Wenliang Xu, Qinran Ding, Yan Zhang, Junqi Zhang, Baocai Zhang, Huan Yu, Chao Li, Longhai Dai, Cheng Zhong, Wenyu Lu, ZhanYing Liu, Feng Li, Hao Song

**Affiliations:** ^1^ Frontier Science Center for Synthetic Biology (Ministry of Education) Key Laboratory of Systems Bioengineering, and School of Chemical Engineering and Technology Tianjin University Tianjin 300072 China; ^2^ State Key Laboratory of Biocatalysis and Enzyme Engineering School of Life Sciences Hubei University Wuhan 430062 China; ^3^ State Key Laboratory of Food Nutrition and Safety key Laboratory of Industrial Fermentation Microbiology, (ministry of education) Tianjin University of Science and Technology Tianjin 300457 China; ^4^ Center for Energy Conservation and Emission Reduction in Fermentation Industry in Inner Mongolia Engineering Research Center of Inner Mongolia for Green Manufacturing in Bio‐fermentation Industry, and School of Chemical Engineering Inner Mongolia University of Technology Hohhot Inner Mongolia 010051 China; ^5^ Haihe Laboratory of Sustainable Chemical Transformations Tianjin 300192 China

**Keywords:** bioelectricity harvest, biohybrid electrode, CO_2_ reduction, interfacial electron transfer, *Shewanella oneidensis*

## Abstract

Interfacial electron transfer between electroactive microorganisms (EAMs) and electrodes underlies a wide range of bio‐electrochemical systems with diverse applications. However, the electron transfer rate at the biotic‐electrode interface remains low due to high transmembrane and cell‐electrode interfacial electron transfer resistance. Herein, a modular engineering strategy is adopted to construct a *Shewanella oneidensis*‐carbon felt biohybrid electrode decorated with bacterial cellulose aerogel‐electropolymerized anthraquinone to boost cell‐electrode interfacial electron transfer. First, a heterologous riboflavin synthesis and secretion pathway is constructed to increase flavin‐mediated transmembrane electron transfer. Second, outer membrane *c*‐Cyts OmcF is screened and optimized via protein engineering strategy to accelerate contacted‐based transmembrane electron transfer. Third, a *S. oneidensis*‐carbon felt biohybrid electrode decorated with bacterial cellulose aerogel and electropolymerized anthraquinone is constructed to boost the interfacial electron transfer. As a result, the internal resistance decreased to 42 Ω, 480.8‐fold lower than that of the wild‐type (WT) *S. oneidensis* MR‐1. The maximum power density reached 4286.6 ± 202.1 mW m^−2^, 72.8‐fold higher than that of WT. Lastly, the engineered biohybrid electrode exhibited superior abilities for bioelectricity harvest, Cr^6+^ reduction, and CO_2_ reduction. This study showed that enhancing transmembrane and cell‐electrode interfacial electron transfer is a promising way to increase the extracellular electron transfer of EAMs.

## Graphical abstract

A modular engineering strategy is adopted to construct a *S. oneidensis*‐carbon felt biohybrid electrocatalytic system, which included increased flavins biosynthesis and secretion, reconstruction of outer‐membrane *c*‐Cyts conductive channel, and bacterial cellulose‐based aerogel‐coated anthraquinone hybrid anode. This biohybrid system improved cell‐electrode interfacial electron transfer rate, which consequently boosted bioelectricity harvest, Cr^6+^ reduction, and CO_2_ reduction.

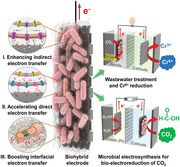

## 1 Introduction

The interfacial electron transfer between electroactive microorganisms (EAMs) and electrodes underlies a wide range of bio‐electrochemical systems (BESs) with diverse applications and emerging potentials in energy production,^[^ ^1^ ^]^ chemical synthesis,^[^ ^2^ ^]^ healthcare diagnostics,^[^ ^3^ ^]^ and environmental detection,^[^ ^4^ ^]^ including microbial fuel cells (MFCs) for simultaneous organic waste biodegradation and bioelectrical harvest,^[^ ^5^ ^]^ unbalanced electro‐fermentation for chemical production,^[^ ^6^ ^]^ microbial electrosynthesis for the bio‐electroreduction of CO_2_,^[^ ^2^, ^7^ ^]^ inorganic nanomaterial biosynthesis,^[^ ^8^ ^]^ and controlled radical polymerization,^[^ ^9^ ^]^ as well as microelectronic devices for biosensors,^[^ ^3b^ ^]^ biobattery capsules,^[^ ^10^ ^]^ and wearable/implantable devices for environmental monitoring and healthcare diagnostics.^[^ ^11^ ^]^ However, the low interfacial electron transfer rate between EAMs and electrodes remains a crucial bottleneck that restricts practical applications of BESs.

*Shewanella oneidensis* is one of well‐studied EAMs, showing great promise as an electroactive biocatalyst in BESs.^[^ ^1^, ^5^ ^]^ Electrons derived from the intracellular catabolism of carbon sources are delivered to the extracellular electrode through two extracellular electron transfer (EET) pathways, including the contact‐based EET pathway based on outer membrane (OM) *c*‐type cytochromes (*c*‐Cyts)^[^ ^12^ ^]^ and the soluble electron shuttle‐mediated EET (MET) pathway based on flavins as electron shuttles or bound cofactors of OM *c*‐Cyts.^[^ ^13^ ^]^ Previous studies have explored a number of synthetic biology and materials engineering approaches to enhance the intracellular electron generation and extracellular electron transfer of *S. oneidensis* for improved bioelectricity production, including regulating substrate metabolism to broaden and strengthen lactate utilization,^[^ ^14^ ^]^ modulating intracellular NADH/NAD^+^ cycling to increase the intracellular releasable electron pool,^[^ ^14^, ^15^ ^]^ enhancing the electron shuttle synthesis and secretion to boost indirect electron transfer,^[^ ^13^, ^16^ ^]^ screening and optimizing *c*‐type cytochromes to accelerate direct electron transfer, and modifying with nanomaterials (such as CNT and GO) to construct conductive biofilms to enhance EET.^[^ ^17^ ^]^ However, the electron transfer rate at the biotic‐electrode interface remains low due to the high transmembrane and cell‐electrode interfacial electron transfer resistances.

**Figure 1:**
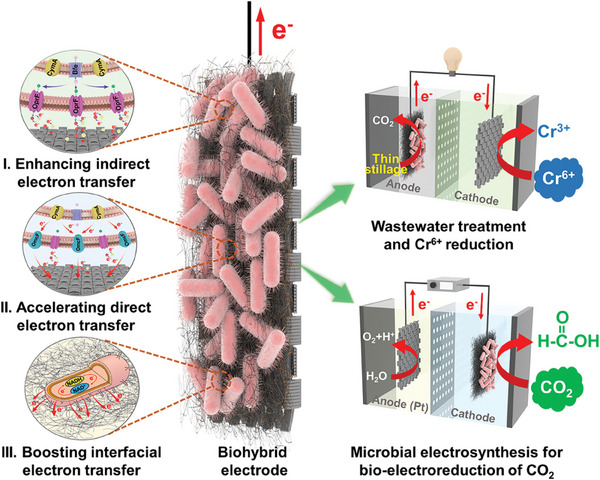
Modular engineering the *S. oneidensis*‐carbon felt biohybrid electrode decorated with bacterial cellulose aerogel‐electropolymerized anthraquinone to boost energy and chemicals production. A modular engineering strategy, including increasing flavins synthesis and secretion, reconstructing OM *c*‐Cyts conductive channel, and constructing bacterial cellulose‐based aerogel coated anthraquinone hybrid anode, was adopted to boost the cell‐electrode interfacial electron transfer rate. i) To address the limitations in the biosynthesis and secretion of endogenous electron shuttle flavins, a heterologous riboflavin synthesis and delivery pathway from *B. subtilis* and *P. aeruginosa* was designed to enhance flavin‐mediated transmembrane electron transfer. ii) To mitigate the issue of the physically insulative cytomembrane of *S. oneidensi*s, outer membrane *c*‐type cytochromes OmcF from *G. sulfurreducens* were screened and optimized using protein engineering strategies to accelerate contact‐based transmembrane electron transfer. iii) To solve the problem of high electron transfer resistance at cell‐electrode interface, an efficient bacterial cellulose aerogel‐coated anthraquinone hybrid electrode was developed to enhance the loading of living cells within the electroactive biofilm on the anode, which boosted cell‐electrode interfacial electron transfer. To evaluate the catalytic performance of the engineered biohybrid electrode in the real‐world applications, bioelectricity harvest from thin stillage, reduction of Cr^6+^ to Cr^3+^, and electrosynthesis of CO_2_ to formate were conducted in the dual‐chamber MFCs.

Herein, to rationally address these issues in the interfacial electron transfer process of BESs (**Figure** **1**), we developed a modular engineering strategy to construct a *S. oneidensis*‐carbon felt biohybrid electrode decorated with bacterial cellulose aerogel‐electropolymerized anthraquinone to boost the cell‐electrode interfacial electron transfer rate. First, to overcome the limited synthesis and transport capacity of endogenous electron shuttle flavins, an efficient riboflavin biosynthesis and secretion pathway from *Bacillus subtilis* and *Pseudomonas aeruginosa* was constructed in the wild‐type (WT) *S. oneidensis* MR‐1 to increase flavin‐mediated transmembrane electron transfer, which led to the biosynthesis of flavins ≈39.9 µm, and the output power density increased from 58.9 ± 4.3 mW m^−2^ to 1100.1 ± 39.7 mW m^−2^. Second, to increase the conductivity of the cytomembrane of *S. oneidensis* cells, we screened and optimized the OM *c*‐Cyt OmcF from *Geobacter sulfurreducens* via protein engineering strategy to accelerate contact‐based transmembrane electron transfer, which increased the single‐cell current output to 201.5 ± 14.6 fA/cell, and the output power density reached 1804.3 ± 97.8 mW m^−2^. Third, to decrease cell‐electrode interfacial electron transfer resistance, bacterial cellulose aerogel coated with electropolymerized anthraquinone was decorated on the *S. oneidensis*‐carbon felt electrode to form a biohybrid electrode, which dramatically enhanced electrochemically active sites and living cell loads in the electroactive biofilm on the anode, thus effectively decreasing cell‐electrode interfacial electron transfer resistance and boosting the interfacial electron transfer rate. As a result, the internal resistance decreased to 42 Ω, 480.8‐fold lower than that of the WT. The maximum output power density reached to 4286.6 ± 202.1 mW m^−2^, 72.8‐fold higher than that of the WT. Lastly, the engineered hybrid electrode exhibited superior abilities for biodegradation of actual thin stillage and bioelectricity harvest, Cr^6+^ reduction, and microbial electrosynthesis of chemicals from CO_2_ reduction. This study showed that enhancing transmembrane and cell‐electrode interfacial electron transfer is a promising avenue to increase the EET rate of EAMs.

## 2 Results

### 2.1 Enhancing Flavins‐Mediated Transmembrane Electron Transfer by Increasing Flavins Synthesis and Secretion

**Figure 2:**
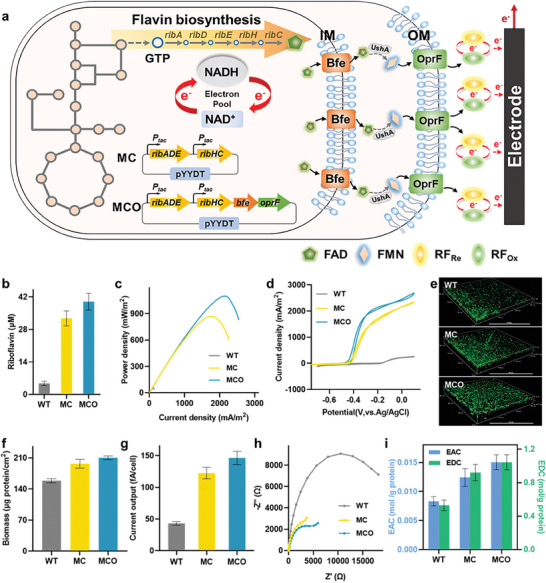
Enhancing flavins‐mediated transmembrane electron transfer by increasing flavins synthesis and secretion. a) Schematic of constructing a flavin biosynthesis pathway to accelerate flavin‐mediated indirect electron transfer, including heterologous expression of the gene cluster *ribADHEC* for flavin synthesis and pore protein OprF and Bfe for flavin transmembrane transport, as well as flavin‐mediated indirect electron transfer pathways. b) The flavin concentrations produced by the WT and recombinant strains (MC and MCO). c) Output power density curves of the WT and recombinant strains (MC and MCO). d) Current‐voltage curves of the WT and recombinant strains (MC and MCO). e) CLSM images of anode biofilms of the WT and recombinant strains (MC and MCO). f) Anode biomass of biofilm formed by the WT and recombinant strains (MC and MCO). g) Single‐cell output current of the WT and recombinant strains (MC and MCO). h) Nyquist plots of EIS spectra for anodes with the WT and recombinant strains (MC and MCO). i) EAC and EDC of the WT and recombinant strains (MC and MCO). Data were presented as mean ± SD (n = 3 biological replicates).

The predominant EET mechanism in *S. oneidensis* involves the utilization of soluble riboflavin or flavin mononucleotides as carriers, which is deemed highly significant, as it accounts for approximately 70–90% of the total electricity transferred.^[^ ^12^, ^13^ ^]^ To enhance flavin‐mediated extracellular electron transfer, the riboflavin synthesis gene cluster derived from *Bacillus subtilis*, including genes of *ribA*, *ribD*, *ribE*, *ribH*, and *ribC*, were employed to express heterologously in *S. oneidensis*, obtaining the recombinant strain MC. Subsequently, to facilitate the secretion of flavin from the recombinant strain MC, the pore protein OprF from *Pseudomonas aeruginosa* and the endogenous transporter Bfe were expressed in the recombinant strain MC (**Figure** **2**a), leading to the recombinant strain MCO. The extracellular flavin synthesized by strains MC and MCO reached 32.7 and 39.9 µm (Figure 2b), respectively, which was 5.8‐ and 7.3‐fold higher than that of the WT strain (4.8 µm), indicating these recombinant strains enabled increase in their riboflavin synthesis and secretion. To study the effect of flavins on EET, the electrophysiological activities of these engineered strains were analyzed, which demonstrated that the highest power density of strains MC and MCO reached 870.1 ± 24.4 mW m^−2^ and 1100.3 ± 39.7 mW m^−2^, respectively, ≈13.8‐ and 17.7‐fold higher than that of the WT strain (58.9 ± 4.3 mW m^−2^) (Figure 2c). Cyclic voltammetry (CV) measurements revealed that the recombinant strains MC and MCO both exhibited prominent flavins reoxidation and reduction peaks at ≈−0.4 V (vs Ag/AgCl) (Figure 2d) corresponding to the flavins‐mediated EET, which suggested that the increased flavin concentration in strains MC and MCO led to higher EET rate than that of the WT strain. Moreover, the peak current density of strain MCO was substantially larger than that of strain MC, suggesting that porin OprF and transporter Bfe accelerated secretion of flavins to further increase the EET rate.

Subsequently, confocal laser scanning microscopy (CLSM) analyses suggested that strains MCO and MC attached on the surface of the carbon felt anode had thickness of 45.8 µm and 50.1 µm (Figure 2e), respectively, ≈1.7‐ and 1.8‐fold of that of the WT strain (27.5 µm). Meanwhile, the quantitative measured biomass of strains MC and MCO attached on the anode surface was 197.0 ± 16.7 µg protein/cm^2^ and 214.2 ± 12.1 µg protein/cm^2^ (Figure 2f), which were 1.2‐ and 1.3‐fold of that of the WT strain (158.6 ± 9.5 µg protein/cm^2^), respectively. This suggested that overexpression of flavin synthesis‐related gene cluster *ribADHEC* in *S. oneidensis* not only accelerated the flavin‐mediated indirect electron transfer, but also promoted biofilm formation, thus further increasing the EET rate. Furthermore, the single cell current of strains MCO and MC was 176.4 ± 10.3 fA/cell and 142.5 ± 9.0 fA/cell (Figure 2g), respectively, which was 4.1‐ and 3.3‐fold of that of the WT strain (43.2 ± 2.9 fA/cell), indicating that flavins improved the transmembrane electron transfer of single cell. Electrochemical impedance spectroscopy (EIS) analysis verified that the internal resistances of strains MCO (≈2.80 kΩ) and MC (≈4.27 kΩ) were apparently lower than that of the WT strain (≈20.19 kΩ) (Figure 2h), demonstrating the overexpression of flavins could reduce the internal resistance of MFCs. To further appraise the effect of the accumulated cells on the electroactivity of biofilm, the electron accepted capacity (EAC) and electron donated capacity (EDC) of these strains were calculated by Equations (1) and (2). Compared to the WT strain, the EAC and EDC values of strains MCO and MC were significantly increased (Figure 2i), which showed improved EET rate and higher electrocatalytic activity. Collectively, our findings demonstrated that enhanced the synthesis and secretion of flavins not only accelerated the flavin‐based EET rate, but also promoted the formation and conductivity of biofilm, thus consequently improving the catalytic ability of the engineered strains.

### 2.2 Accelerating Contacted‐Based Transmembrane Electron Transfer by Reconstructing OM *c‐*Cyts Conductive Channel

**Figure 3:**
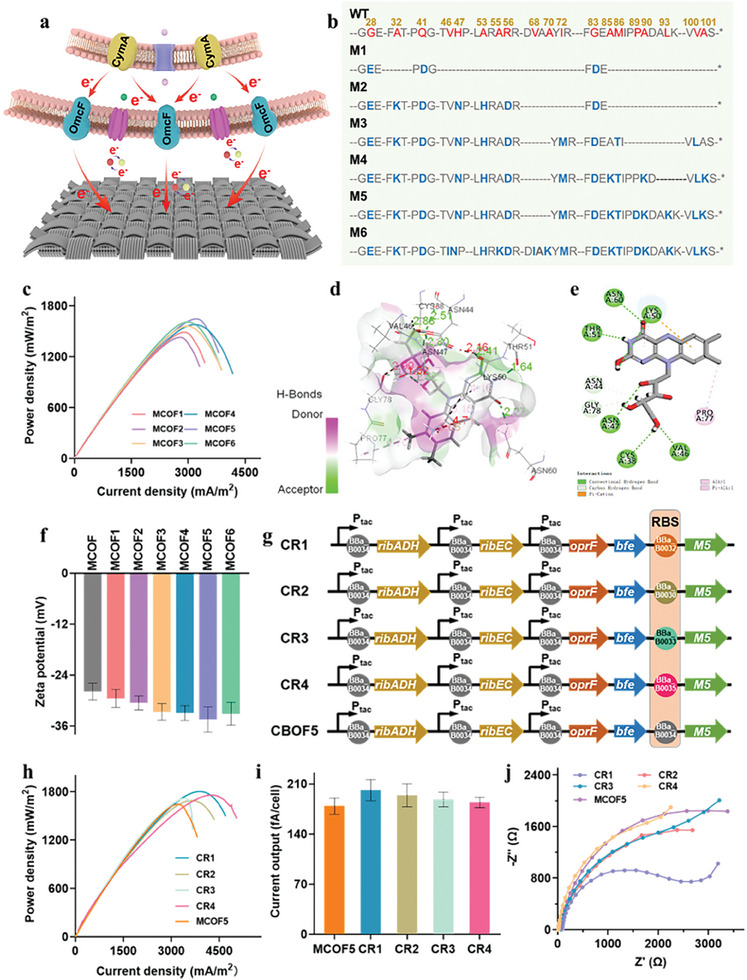
Accelerating contacted‐based transmembrane electron transfer by reconstructing OM *c‐*Cyts conductive channel. a) Schematic of the outer‐membrane *c*‐Cyts and riboflavin promoting transmembrane electron transfer from cell to anode. b) Amino acid sequences and mutation sites of the WT and mutant OmcF M1‐M6. c) Power densities of the mutant strains MCOF1‐6. d) The interaction between *c*‐Cyt OmcF M5 and riboflavin molecule. e) The 2D display of the interaction between the residues of OmcF M5 and RF molecule. f) Zeta potentials of strains MCOF and MCOF1‐6. g) Plasmids of the recombinant strains MCOF5 and CR1‐4 that included the assemblage of genes (*ribADEHC*, *bfe*, *oprF*, and *omcF* M5) and different RBSs (BBa_B0030, BBa_B0032, BBa_B0033, BBa_B0034, BBa_B0035, iGEM). h) Output power density curves of strains MCOF5 and CR1‐4. i) Single‐cell output current of the recombinant strains MCOF5 and CR1‐4. j) EIS analysis of the recombinant strains MCOF5 and CR1‐4. Data were presented as mean ± SD (n = 3 biological replicates).

To accelerate contacted‐based transmembrane electron transfer, four conductive OM *c*‐Cyts, including OmcB, OmcE, and OmcF from *G. sulfurreducens* PCA, and MtrC from *S. oneidensis* MR‐1, were further overexpressed in strain MCO to reconstruct the OM *c*‐Cyts conductive channel (**Figure** **3**a), obtaining four engineered strains MCOF, MCOE, MCOB, and MCOC, respectively. Raman spectroscopy analysis confirmed that the four signature bands of *c*‐Cyts at 749, 1128, 1312, and 1584 cm^−1^ were detected in these engineered strains (Figure S1a, Supporting Information). The intensities of these *c*‐Cyts bands in the engineered strains were greater compared to the control strain MCO. The UV‐visible spectroscopy also substantiated that all the engineered strains emerged a *c*‐Cyts characteristic absorption peak at 418 nm, and the peak intensities were higher than that of strain MCO (Figure S1b, Supporting Information). These results verified that the engineered strains contained higher amounts of *c*‐Cyts anchored onto OM, which were expected to promote the transmembrane electron transfer. Electrochemical characterization showed that the power density of strain MCOF reached 1466.2 ± 46.1 mW m^−2^, which was higher than those of strains MCOE (1410.6 ± 31.6 mW m^−2^), MCOB (1320.6 ± 65.3 mW m^−2^), MCOC (1377.5 ± 62.9 mW m^−2^), and MCO (1100.3 ± 39.7 mW m^−2^) (Figure S2a, Supporting Information). These conclusions suggested that heterogeneously expressed OmcF presented a stronger ability to facilitate transmembrane electron transfer. Similarly, the CV curves revealed that the peak current density of strain MCOF was remarkably higher than those of other engineered strains (Figure S2b, Supporting Information), manifesting that a greater number of redox‐active species were involved in the transmembrane electron transfer process of strain MCOF.

To further improve the transmembrane electron transfer rate of *c*‐Cyt OmcF, rational protein engineering strategy was adopted to obtain a mutant OmcF with high EET rate. We first determined the protein structure of OmcF, then performed sequence alignment using the PROSS (Protein Redesign through Optimized Side‐chain Sampling) and Consensus Finder databases to rapidly predict potential mutant sites.^[^ ^18^ ^]^ This approach allowed us to disregard the conserved sites, as they are less likely to benefit the protein property from mutation. The PROSS method combines phylogenetic analysis with Rosetta atomistic design calculations, while it fully conserves the amino acid sequence and sidechain conformations at the active site and eliminates mutations that are rare among homologs or predicted to destabilize the native state according to Rosetta atomistic calculations. Based on the structural model of OmcF, the PROSS analysis made it possible to predict the beneficial mutation sites combinations for enhancing the transmembrane electron transfer rate. Thus, beneficial mutations of the gene *omcF*, including *omcF1*, *omcF2*, *omcF3*, *omcF4*, *omcF5*, and *omcF6*, were generated by PROSS (Figure 3b) and inserted into strain MCO to obtain strains MCOF1, MCOF2, MCOF3, MCOF4, MCOF5, and MCOF6, respectively. Among these strains, strain MCOF5 obtained the highest maximum power density of 1643.8 ± 74.6 mW m^−2^, which was higher than that of strains MCOF1 (1485.7 ± 72.2 mW m^−2^), MCOF2 (1536.3 ± 66.9 mW m^−2^), MCOF3 (1559.4 ± 84.1 mW m^−2^) and MCOF4 (1577.9 ± 82.4 mW m^−2^), and MCOF6 (1609.1± 93.5 mW m^−2^) (Figure 3c).

To further understand the mechanism of enhanced EET performance by the mutant strains, Protein‐Docking simulations of the interaction between mutant OM *c*‐Cyt OmcF M5 and flavin were analyzed. The result showed the distance between the mutant OmcF M5 and flavin molecule decreased from 1.95 Å to 1.32 Å (Figure 3d; Figure S3, Supporting Information), and the interaction between mutant M5 and riboflavin was enhanced (Figure 3e; Figure S4, Supporting Information), which facilitated the electrons transfer from OmcF to flavin, thus accelerating the flavin‐mediated indirect electron transfer. Meanwhile, the surface zeta potential analysis results showed that introducing a large number of charged residues (E28, D41, D56, D83, and D89) into mutant OmcF enhanced the surface polarity and interaction between cell and anode (Figure 3f).

Subsequently, to better control the expression of the mutant OmcF, four different expression intensities ribosome binding site (RBS) sequence were identified and then integrated in strain MCOF5, obtaining recombinant strains CR1, CR2, CR3, and CR4, respectively (Figure 3g). Among them, the maximum output power density and single‐cell current output of strain CR1 reached 1804.3 ± 97.8 mW m^−2^ (Figure 3h) and 201.5 ± 14.6 fA/cell, respectively, which were observably higher than those of the other strains (Figure 3i), indicating that the optimal expression of the mutant OmcF M5 could effectively improve the transmembrane electron transfer in *S. oneidensis*. EIS analysis also confirmed strain CR1 possessed the lowest internal resistance (≈1861 Ω) (Figure 3j), indicating that optimizing the expression intensity of the mutant OmcF endowed strain CR1 the efficient transmembrane electron transfer channel, thus reducing internal resistance of MFCs and enhancing the current output. Collectively, reconstructing OM *c*‐Cyts conductive channel could further accelerate contacted‐based transmembrane electron transfer of the engineered *S. oneidensis*.

### 2.3 Boosting Cell‐Electrode Interfacial Electron Transfer by Constructing Biohybrid Electrode Decorated with Bacterial Cellulose Aerogel and Electropolymerized Anthraquinone

**Figure 4:**
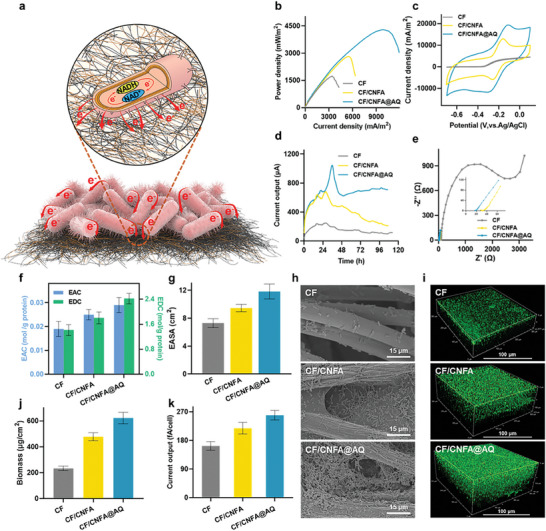
Boosting cell‐electrode interfacial electron transfer by constructing *S. oneidensis*‐carbon felt biohybrid electrode decorated with bacterial cellulose aerogel‐electropolymerized anthraquinone. a) Schematic of interfacial electron transfer process after decoration of CNFA and AQ on the surface of the carbon felt anode. b) Power density curves of strain CR1 equipped with the CF and decorated anodes (CF/CNFA and CF/CNFA@AQ). c) CV curves of strain CR1 equipped with the CF and decorated anodes (CF/CNFA and CF/CNFA@AQ). d) Current output of strain CR1 equipped with the CF and decorated anodes (CF/CNFA and CF/CNFA@AQ). e) EIS analysis of strain CR1 equipped with the CF and decorated anodes (CF/CNFA and CF/CNFA@AQ). f) EAC and EDC of strain CR1 equipped with the CF and decorated anodes (CF/CNFA and CF/CNFA@AQ). g) Electrochemically active surface area of the CF and decorated anodes (CF/CNFA and CF/CNFA@AQ). h) SEM images of colony morphology of strain CR1 equipped with the CF and decorated anodes (CF/CNFA and CF/CNFA@AQ). i) CLSM images of colony morphology of strain CR1 equipped with the CF and decorated anodes (CF/CNFA and CF/CNFA@AQ). j) Analysis of biomass of strain CR1 equipped with the CF and decorated anodes (CF/CNFA and CF/CNFA@AQ). k) Single‐cell output current of strain CR1 equipped with the CF and decorated anodes (CF/CNFA and CF/CNFA@AQ). Data were presented as mean ± SD (n = 3 biological replicates).

Increasing synthesis and secretion of flavins and reconstructing OM *c*‐Cyts conductive channel have prominently facilitated the transmembrane electron transfer of *S. oneidensis*, however, the high cell‐electrode interfacial electron transfer resistance still restricted electrons transfer from cells to the anode. Herein, to improve cell‐electrode interfacial electron transfer, an efficient *S. oneidensis*‐carbon felt biohybrid anode with decoration of bacterial cellulose aerogel coated with electropolymerized anthraquinone was constructed. A hierarchically porous carbon nanofiber aerogel (CNFA) was prepared from bacterial cellulose (Figure S5, Supporting Information), which was further utilized to decorate the carbon felt (CF) anode (named as CF/CNFA anode). Subsequently, the decorated CF/CNFA anode was electropolymerized with anthraquinone (AQ), resulting in the CF/CNFA@AQ anode (**Figure** **4**a; Figure S6, Supporting Information). Electrochemical characterization displayed that the engineered strain CR1 equipped with the CF/CNFA@AQ anode showed the maximum power density of 4286.6 ± 202.1 mW m^−2^ (Figure 4b), which was 2.4‐ and 1.5‐fold higher than those of the CF (1804.3 ± 97.8 mW m^−2^) and CF/CNFA anodes (2812.6 ± 99.5 mW m^−2^), respectively. The CV analysis and output current from chronoamperometry showed that the CF/CNFA@AQ anode exhibited the highest catalytic current density (Figure 4c,d), suggesting that the rate of interfacial electron transfer could be efficaciously boosted by the decoration with CNFA and AQ.

To expound the mechanism behind the improved interfacial electron transfer rate, EIS characterization was performed to reveal that the MFC equipped with the CF/CNFA@AQ anode presented the lowest internal resistance of 45 Ω, which was 41.4‐ and 4.1‐fold lower than those of the CF (1861 Ω) and CF/CNFA anodes (185 Ω), respectively (Figure 4e), indicating that the decoration of the anode with CNFA and AQ effectively decreased the internal resistance of electron transfer from the *S. oneidensis* cells to the anode. Furthermore, the decreased internal resistance resulted in 1.5‐fold increase in EAC and a 1.7‐fold increase in EDC, compared to the native anode (Figure 4f). The decreased internal resistance and the increased EAC and EDC suggested that the decorated anodes possessed higher electron extracting and collecting ability by forming efficient electron transfer channels.

To further elucidate the specific mechanisms of the decreased cell‐electrode interfacial electron transfer resistance by simultaneous decoration with CNFA and AQ, the electrochemically active surface area (EASA) of the anode CF/CNFA@AQ was determined to be 11.83 cm^2^ (Figure 4g) according to the Matsuda's equation (Equation (3)), which was 0.62‐ and 0.25‐fold higher than those of the CF (7.31 cm^2^) and CF/CNFA anodes (9.46 cm^2^), respectively, suggesting that the simultaneous decoration with CNFA and AQ endowed CF/CNFA@AQ anode with more electrochemically active sites. Scanning electron microscope (SEM) and CLSM analyses further manifested that more viable cells were loaded on the surface of the CF/CNFA@AQ anode to form a denser and thicker electroactive biofilm compared to the CF/CNFA and CF anodes (Figure 4h,i). Meanwhile, the biomass on CF/CNFA@AQ anode was estimated to be 622.4 ± 44.2 µg cm^−2^, which was markedly higher than those of the CF (233.1 ± 17.3 µg cm^−2^) and CF/CNFA anodes (477.9 ± 30.5 µg cm^−2^) (Figure 4j). In addition, the surface area, pore size, and hydrophilicity of the decorated electrode were greatly improved (Figures S7 and S8, Supporting Information), which further explained the increase in the coverage of cells on the surface of decorated electrodes. Moreover, the current output per single cell of strain CR1 equipped with CF/CNFA@AQ anode reached 236.4 ± 14.8 fA/cell (Figure 4k), which was 1.35‐ and 2.17‐fold higher than that of the CF/CNFA and native CF anodes, respectively, suggesting that the decorated electrode could effectively boost the abiotic/biotic interfacial electron transfer rate at single‐cell level, thereby promoting the EET rate of BES. These results confirmed that more electrochemically active sites and more living cells were loaded onto the decorated anodes, which effectively decreased cell‐electrode interfacial electron transfer resistance and boosted the abiotic/biotic interfacial electron transfer rate.

In all, a modular engineering strategy, including increasing flavins synthesis and secretion, reconstructing OM *c*‐Cyts conductive channel, and decoration of the biohybrid electrode by bacterial cellulose aerogel and electropolymerized anthraquinone, was adopted to boost cell‐electrode interfacial electron transfer rate, which significantly decreased internal resistances to 42 Ω, 480.8‐fold lower than that of the WT *S. oneidensis* MR‐1 (Figure S9a, Supporting Information). The single‐cell current and maximum output power density were increased to 236.4 ± 14.8 fA/cell (Figure S9b, Supporting Information) and 4286.6 ± 202.1 mW m^−2^, 4.5‐ and 72.8‐fold higher than that of the WT strain, respectively (Figure S9c, Supporting Information). Moreover, the output power density, the maximum current output per single cell, and the internal resistance of each module, including MCO‐CF, SO (expressing only the optimized mutant *omcF* M5)‐CF and WT‐CF/CNFA@AQ, outcompeted that of the control (WT‐CF). Specifically, the power densities of MCO‐CF, SO‐CF and WT‐CF/CNFA@AQ reached 1100.0 ± 39.7 mW m^−2^, 536.2 ± 22.4 mW m^−2^, and 2053.7 ± 92.6 mW m^−2^ (Figure S10a, Supporting Information), respectively, ≈17.7‐, 8.1‐, and 33.9‐fold higher than that of the WT strain (58.9 ± 4.3 mW m^−2^). The maximum current output per single cell of MCO‐CF, SO‐CF, and WT‐CF/CNFA@AQ reached 176.4 ± 10.3 fA/cell, 67.4 ± 5.7 fA/cell, and 214.5 ± 5.7 fA/cell (Figure S10b, Supporting Information), respectively, which were much higher than that of WT‐CF (43.2 ± 2.9 fA/cell). The internal resistance of MCO‐CF, SO‐CF, and WT‐CF/CNFA@AQ were detected to be 2.80, 5.68, and 0.55 kΩ (Figure S10c, Supporting Information), respectively, significantly lower than that of WT‐CF (20.19 kΩ).

### 2.4 Applying the Biohybrid Electrode in Real‐World Scenarios for Energy and Chemicals Production

To further evaluate the catalytic performance and stability in the real‐world scenarios, we constructed the biohybrid electrode with the engineered *S. oneidensis* strain CR‐1 (CF/CNFA@AQ‐CR1) in the dual‐chamber MFC, in which various applications were performed, including bioelectricity harvest from actual thin stillage, Cr^6+^ reduction, and electrocatalytic CO_2_ reduction.

**Figure 5:**
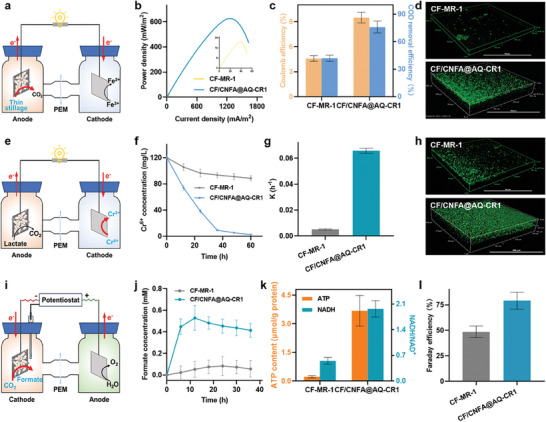
Application of the constructed biohybrid electrode in real‐world scenarios for energy and chemicals production. a) Schematic diagram of electricity recovering from thin stillage using constructed BES. b) Power density curves of the engineered biohybrid electrode CF/CNFA@AQ‐CR1 and control. c) The Coulombic efficiency and COD removal efficiency of the engineered biohybrid electrode CF/CNFA@AQ‐CR1 and control in thin stillage. d) CLSM images of the engineered biohybrid electrode CF/CNFA@AQ‐CR1 and control. e) Schematic diagram of Cr^6+^ degradation using constructed BES. f) The Cr^6+^ concentrations of the engineered biohybrid electrode CF/CNFA@AQ‐CR1 and control in MFCs under anaerobic conditions. g) Cr^6+^ degradation kinetic constant of the engineered biohybrid electrode CF/CNFA@AQ‐CR1 and control in MFCs. h) CLSM images of the engineered biohybrid electrode CF/CNFA@AQ‐CR1 and control. i) Schematic diagram of CO_2_ reduction to produce formate by electrosynthesis using the engineered biohybrid electrode. j) The formate concentrations of the engineered biohybrid electrode CF/CNFA@AQ‐CR1 and control. k) The ATP concentrations and NADH/NAD^+^ ratio of the engineered biohybrid electrode CF/CNFA@AQ‐CR1 and control. l) The Faraday efficiency of the engineered biohybrid electrode CF/CNFA@AQ‐CR1 and control. Data were presented as mean ± SD (n = 3 biological replicates).

Organics‐rich thin stillage is an essential by‐product of the liquor brewing industry, which can cause severe water pollution when directly released into the environment.^[^ ^14^, ^19^ ^]^ The electroactive cells could recover bioelectricity from the organics in the thin stillage to achieve sustainable bioelectricity production. To evaluate the potential and stability of the engineered biohybrid electrode in recovering electrical energy from actual thin stillage, the constructed biohybrid electrode CF/CNFA@AQ‐CR1 was incubated in MFC fed with actual thin stillage (**Figure** **5**a). The maximum output power density of the engineered biohybrid electrode CF/CNFA@AQ‐CR1 reached 625.3 ± 25.7 mW m^−2^ (Figure 5b), 29.4‐fold higher than that of the control (CF‐*S.oneidensis* MR‐1) (21.3 ± 3.6 mW m^−2^). Subsequently, the energy recovery capacity from thin stillage was measured by the chemical oxygen demand (COD) removal efficiency. The result showed that the biohybrid electrode CF/CNFA@AQ‐CR1 could efficiently remove up to 76.0 ± 6.5% of COD (Figure 5c), which was much higher than that of the control (42.1 ± 3.3%). Furthermore, the electron recovery capacity of the biohybrid electrode was characterized by Coulombic efficiency (C_E_) (Figure 5d). The C_E_ of the engineered biohybrid electrode CF/CNFA@AQ‐CR1 was 9.5%, which was 2.1‐fold of the control (4.6%). CLSM analysis showed that the CF/CNFA@AQ‐CR1 hybrid electrode formed a thicker and denser biofilm than that of the control (Figure 5e), indicating that the CF/CNFA@AQ‐CR1 biohybrid electrode possessed higher stability in the actual thin stillage environment. These results showed that the constructed biohybrid electrode was a prospective biocatalytic system to recovery electrical energy efficiently from actual thin stillage.

Chromium is a toxic heavy metal widely used in industries such as welding, tanning, and electroplating. Cr^6+^, with carcinogenic and teratogenic, has a high solubility, fluidity and toxicity, which was challenging to be reduced in the natural state, and caused serious deterioration of the ecosystem when released into the natural environment.^[^ ^20^ ^]^ Therefore, the biohybrid electrode CF/CNFA@AQ‐CR1was utilized to explore the performance in Cr^6+^ reduction (Figure 5f). As expected, the biohybrid electrode CF/CNFA@AQ‐CR1 exhibited faster Cr^6+^ reduction rate than that of the control. Especially, CF/CNFA@AQ‐CR1 could reduce Cr^6+^ ≈96% within 48 h (Figure 5g), which was ≈2.4‐fold higher than that of control (40%). To further explored Cr^6+^ reduction kinetics, we used Pseudo‐First order kinetics equation to depict the Cr^6+^ removal rate (Equation (4)). The calculated kinetic constant of the biohybrid electrode CF/CNFA@AQ‐CR1 was 0.0316 h^−1^, which was 1.8‐fold higher than that of the control (0.0175 h^−1^) (Figure 5h), indicating that the constructed biohybrid electrode has higher Cr^6+^ reduction ability. Subsequently, CLSM characterization revealed that there were more living cells in CF/CNFA@AQ‐CR1, which further verified engineered biohybrid electrode had higher stability than that of the control.

The excessive emission of CO_2_ into the atmosphere has led to severe climate change, which becomes a pressing environmental issue of global concern.^[^ ^21^ ^]^ Consequently, developing an efficient methodology to convert CO_2_ into value‐added chemicals is an attractive approach in addressing such concerns.^[^ ^22^ ^]^ To assess the catalytic performance of CO_2_ reduction, the constructed three‐electrode double chamber BES was used to reduce CO_2_ to formate, which maintained the constant potential of −0.7 V (vs Ag/AgCl) at the cathode (Figure 5i). As predicted, the constructed biohybrid electrode CF/CNFA@AQ‐CR1 with bicarbonate as the sole carbon source yielded higher formate concentration of 0.58 ± 0.11 mm within 30 h (Figure 5j), ≈3.7‐fold higher than that of the control, revealing that engineered *S. oneidensis* CR1 could uptake electrons from the constructed biohybrid electrode and reduce CO_2_ to formate. Meanwhile, the increase in intracellular ATP content and NADH/NAD^+^ ratio indicated that the engineered strain *S. oneidensis* CR1 can quickly uptake electrons from the electrode, and transform the electrons into intracellular reducing equivalents (Figure 5k). Moreover, the Faraday efficiency (calculated by Equation (5)) of the engineered biohybrid electrode reached 79.2% (Figure 5l), ≈1.6‐fold higher than that of the control (48.6%), suggesting the engineered biohybrid electrode CF/CNFA@AQ‐CR1 has higher electron transfer efficiency for chemical synthesis.

## 3 Discussion

The electron transfer rate at the biotic‐electrode interface remains low due to the high transmembrane and cell‐electrode interfacial electron transfer resistances. To rationally address this issue in the interfacial electron transfer process of BESs, a modular engineering strategy, including increasing flavins synthesis and secretion, reconstructing OM *c*‐Cyts conductive channel, and decoration of the biohybrid electrode by bacterial cellulose aerogel and electropolymerized anthraquinone, was adopted to boost cell‐electrode interfacial electron transfer rate in this study.

Three scientific novelties and insights of this study are summarized as follows: 1) To overcome the limited synthesis and transport of flavins (the endogenous electron shuttles of *S. oneidensis*), flavins‐mediated transmembrane electron transfer was enhanced by increasing flavins synthesis and secretion. Our findings demonstrated that the enhanced synthesis and secretion of flavins not only accelerated the flavin‐based EET rate, but also promoted the formation and conductivity of biofilm, which consequently enhanced power generation of the engineered strains. 2) To increase the conductivity of the cytomembrane of *S. oneidensis*, rational protein engineering strategy was adopted to obtain a mutant OmcF with high EET rate. To the best of our knowledge, this protein engineering strategy was a first attempt for the optimization of outer membrane *c*‐type cytochromes for EET enhancement. Our results indicated the optimal expression of the mutant OmcF M5 could effectively reduce the transmembrane resistance and enhance the transmembrane electron transfer in *S. oneidensis*. 3) To form a biohybrid electrode, bacterial cellulose aerogel coated with electropolymerized anthraquinone was decorated on the *S. oneidensis*‐carbon felt electrode, which dramatically enhanced electrochemically active sites and living cell loads in electroactive biofilm on the anode, thus effectively decreasing cell‐electrode interfacial electron transfer resistance and boosting the interfacial electron transfer rate. For the first time, the bacterial cellulose aerogel and electropolymerized anthraquinone were simultaneously used to decorate the *S. oneidensis*‐carbon felt electrode to form a biohybrid electrode. As a result, the maximum output power density of the biohybrid electrode reached 4286.6 ± 202.1 mW m^−2^, 72.8‐fold higher than that of the WT *S. oneidensis* MR‐1, which exhibited superior power density over previous works (see Table S5, Supporting Information).

Furthermore, the engineered biohybrid electrode exhibited superior abilities for biodegradation of actual thin stillage and bioelectricity harvest, Cr^6+^ reduction, and microbial electrosynthesis of formate from CO_2_ reduction. This study showed that enhancing transmembrane and cell‐electrode interfacial electron transfer is a promising approach to increase the EET rate of EAMs.

## 4 Experimental Section

### Plasmid Construction and Bacterial Culture

The strains and plasmids utilized in this investigation were detailed in Tables S1 and S2 (Supporting Information). All constructed plasmids were conducted in the auxotrophic *Escherichia coli* WM3064 strain, supplemented with 2,6‐diaminopimelic acid (DAP) (100 µg mL^−1^) and kanamycin (50 µg mL^−1^) as necessary. To achieve multigene editing in *S. oneidensis*, the expression vector pYYDT was employed. Plasmids were initially transformed into the *E. coli* WM3064, and subsequently transferred into *S. oneidensis* via conjugation. Detailed sequences of relevant genes can be found in Table S3 (Supporting Information).

### Bio‐Electrochemical Systems Operation

Secondary seed solution was harvested by centrifugation (8000 rpm, 5 min). Cell pellets were then adjusted to an optical density of 0.7 with anolyte solution, consisting of M9 buffer, LB broth (5%), lactate (20 mm), MgSO_4_ (1 mm), CaCl_2_ (0.1 mm), NaOH (4 mm), IPTG (1 mm), and kanamycin (50 µg mL^−1^) as required, and then filled with anolyte solution to 120 mL. Simultaneously, 120 mL catholyte, containing K_3_[Fe(CN)_6_] (50 mm), KH_2_PO_4_ (50 mm), and K_2_HPO_4_ (50 mm), were added to the cathode chamber. Nafion117 membranes separated the anodic and cathodal chambers. The anode was made of decorated/native carbon felt (1 × 1 cm), and the cathode was native carbon felt in size of 2.5 × 3 cm. Subsequently, the MFCs were sealed after 15 min of purging with pure nitrogen to ensure anaerobic conditions.

### Electrochemical Characterizations

The MFCs described above were connected with an external resistor (2000 Ω) to enable voltage measurement and recording. Three‐electrode system was utilized for cyclic voltammetry analysis. The scanning rate was set at 1 mV s^−1^, and the scanning was repeated for four cycles in the potential window of – 0.7–+ 0.1 V. Power values were obtained through linear sweep voltammetry assay with a scan rate of 0.1 mV s^−1^. Current output curves were recorded under a voltage of +0.2 V (vs Ag/AgCl). Electrochemical impedance spectroscopy analyses were conducted utilizing the frequency response analyzer connected to a potentiostat.

### Measurement of Flavins

Overnight cultures of *S. oneidensis* were inoculated into LB broth (1% inoculum) supplemented with antibiotic and isopropyl‐β‐d‐thiogalactoside (IPTG) as inducers. After ≈12 h of growth, flavin content was determined utilizing High‐Performance Liquid Chromatography with the UV detector. The sample underwent filtration and analysis using a reverse‐phase C18 column. The flow rate was set at 0.1 mL min^−1^, the column temperature was 30 °C, and the injection volume at 20 mL.^[^ ^13c^ ^]^

### Measurement of Anode Biomass

To measure the anode protein concentration, the samples were first cut carefully and placed in centrifuge tube and rinsed with PBS solution. The samples were vortexed for 3 min and incubated at 95 °C for 40 min to facilitate cell lysis. The samples were determined with BCA protein assay kit (Beyotime, China) after cooled to RT.

### Confocal Imaging of Biofilm

Nikon A1R+ confocal laser scanning microscopy was utilized to capture confocal images. The anodes from MFCs biofilms were first washed in PBS (0.1 m) to wipe off the medium, and then stained with LIVE/DEAD BacLightTM bacterial viability kit. The laser wavelengths were 488 and 561 nm for further analysis.

### Electron Exchange Capacity Analysis

Electrochemical characterizations were carried out using a three‐electrode system. The working electrode potentials were +0.61 V for the EDC and −0.49 V for the EAC. EDC and EAC values were determined by integrating the oxidative and reductive current, respectively, as shown in the following equations:^[^ ^17a^ ^]^

(1) $$\mathrm{EAC}\;=\frac{\int_{0}^{t}I_{red}d_{t}}{Fm_{protein}}$$

(2) $$\mathrm{EDC}\;=\frac{\int_{0}^{t}I_{ox}d_{t}}{Fm_{protein}}\;$$

where I_red_ was the reductive current (A), I_ox_ was the oxidative current (A), F was Faradic constant (96 485 C mol^−1^), and m_protein_ represented the mass of anode protein (g).

### Preparation and Analysis of Decorated Electrodes

The bacterial cellulose was first cut into small pieces, neutralized with deionized water, followed by vacuum freeze dried to obtain bacterial cellulose aerogels. The bacterial cellulose aerogels were then pyrolyzed under a flowing argon atmosphere. The heating rate was set to 2 °C min^−1^ to 500 °C, held for 1 h, and then increased to 5 °C min^−1^ to reach temperatures between 600–1100 °C, held for 1 h, to yield black carbon nanofiber aerogels (CNFA).^[^ ^17c^ ^]^ The CNFA aerogels were then pulverized into powders and mixed with 1wt% poly(tetrafluoroethylene), then coated on the carbon felt (CF) electrode surface and dried at 100 °C for 3 h to fabricate the CF/CNFA anodes.

Electrochemical polymerization of anthraquinone (AQ) on the CF/CNFA anodes was then carried out in a three‐electrode system. The aforesaid CF/CNFA anodes were subsequently transferred into an electrolyte solution, which containing PBS (100 mm, pH 7.0), NaCl solution (0.1 m), and AQ (1 mm). AQ then electropolymerized through cyclic voltammetry with 60 repeated cycles in a potential range from −1.0 to +1.5 V with rate of 50 mV s^−1^. Finally, the prepared anode was rinsed three times with PBS to wipe off any free anthraquinone, which named as CF/CNFA@AQ anode.

### Pore Size and Surface Area Measurement

The Brunauer–Emmett–Teller (BET) surface area and Barrett–Joyner–Halenda (BJH) pore size of the anodes were measured utilizing a 3H‐2000PS1 instrument.

### Water Contact Angle Assay

The hydrophobicity and hydrophilicity of the electrodes were assayed with a JC2000A instrument (Powereach Co., China). To ensure higher accuracy, the contact angle value was assayed at least five independently.

### Electrochemically Active Surface Area (EASA) Analysis

The anolyte solution consisted of K_3_[Fe(CN)_6_] (5 mm) and Na_2_SO_4_ (0.2 m). The CV was conducted in a potential from −0.4 to 1.0 V, the scan rate is 10 mV s^−1^. Then, the EASA was gained utilizing the next Matsuda's equation:^[^ ^17^, ^23^ ^]^

(3) $$i_{p}=\frac{0.4464\;*\;10^{-3}n^{\frac{3}{2}}F^{\frac{3}{2}}AD^{1/2}C_{R}v^{1/2}}{{\left(RT\right)}^{1/2}}$$

where n was transferred electrons number (here n = 1), R was gas constant (8.314 J/mol·K), F was Faradic constant (96 487 C mol^−1^), T was temperature (303 K), v was scan rate s (0.05 V/s), C_R_ was initial ferrocyanide concentration (5 mm), D was diffusion coefficient of the K_3_[Fe(CN)_6_] solution (3.7 × 10^−6^ cm^2^/s), A was EASA value (cm^2^).

### The Measurement of Coulombic Efficiency

The coulombic efficiency *C_E_* was calculated as follows:

(4) $$C_{E}=\frac{M_{S}\int_{0}^{t_{b}}Id_{t}}{Fb_{ES}V_{An}\Delta C}$$

where M_S_ is the substrate (lactate) molecular weight (g/mol), I represents current (A), F is Faradic constant (98485 C/mol), t_b_ represents the time of a cycle (s), b_ES_ represents electrons number produced per mole of substrate (here b = 4), V_An_ represents the anolyte volume (L), and Δc represents the change of substrate concentration (g/L).^[^ ^17b^ ^]^

### Raman Spectroscopic Analyses of *c‐*Cyts

3 mL secondary seed culture were collected through centrifugation (10,000 rpm, 5 min), then resuspended in ultrapure water. The suspensions were spread on a slide and analyzed using a Confocal Raman Microscope (Horiba, France) with a laser of 532 nm.^[^ ^17b^ ^]^

### Scanning Electron Microscope Characterizations

The morphology of cells attached on the surface of anode were characterized using a FESEM. Prior to the SEM observation, the anodes were soaked in glutaraldehyde (2.5%) for 8 h. The samples were then treated with gradient alcohol (30%, 50%, 70%, 80%, 90%, and 100%) solutions, and finally vacuum freeze dried. The dried samples were cut into 0.5 cm × 0.5 cm pieces, followed by coated with gold before the SEM analysis.

### Recovery of Electricity from Thin Stillage

To verify the electricity recovery from real thin stillage, the engineered strain CR1 was cultured overnight and then inoculated into real thin stillage (high‐temperature sterilization) with decorated electrode CF/CNFA@AQ as working anode. An equal volume of catholyte was added to the cathode chamber, which equipped a CF electrode as cathode, followed by connected to the electrochemical workstation.^[^ ^19^ ^]^

### Calculation of Cr^6+^ Removal Kinetics

The linear relationship between ln (C_t_/C_0_) and time (t) indicates that the Cr^6+^ reduction follows pseudo‐first‐order kinetics, as described by the following equation:^[^ ^24^ ^]^

(5) $$\ln\;\left(C_{t}\;/C_{0}\;\right)=\;-\mathrm{kt}$$

where C_t_ (mg/L) represents the concentration of Cr^6+^ at time t, C_0_ represents the initial concentration of Cr^6+^ (mg/L), t represents the reaction time (s), k represents the kinetic rate constant.

### Faradaic Efficiency Measured

The Faradic efficiency of electrosynthesis of CO_2_ to formate was gained by the following formula:^[^ ^25^ ^]^

(6) $$\eta \;=\frac{b\;\times \;\mathrm{nHCOOH}\;\times \;F}{Q}\times 100\%$$

where b represented exchanged electrons number for CO_2_ reduction (b = 2 for formate), n_HCOOH_ represented the amount of formate produced (mol), F represented Faradic constant (96 485 C mol^−1^), *Q* represented total consumed electrons (C) number during the electrosynthesis process.

### Electrosynthesis of Carbon Dioxide to form Formate

Three‐electrode double‐chamber microbial electrosynthesis system was utilized for CO_2_ fixation. The setup consisted of reference electrode (KCl‐saturated Hg/Hg_2_Cl_2_), counter electrode (Platinum electrode), and working electrode: CF/CNFA@AQ‐CR1 biohybrid. The Nafion 117 proton membrane was used to separate anode chamber and cathode chamber. The system assembly was operated in an anaerobic workstation. 8 mL NaHCO_3_ (1 m, sterilized with a 0.22 µm filter and deoxygenated by N_2_ bubbling for 30 min) was added to the 112 mL cathode solution and then bubbled with N_2_. The anode solution contained NaCl (50 mm) and H_2_SO_4_ (1 mm). Electrochemical workstation was used to maintain a constant potential of −0.7 V (vs Ag/AgCl) at cathode.^[^ ^25^, ^26^ ^]^

The formate concentration was measured using HPLC equipped with an HPX‐87H (BIO‐RAD Aminex, 300 mm × 7.8 mm) column. H_2_SO_4_ (4 mm) was utilized as mobile phase, the flow rate was 0.4 mL min^−1^. The analysis was carried out at 50 °C, with an automatic injection volume of 10 µL.

## Conflict of Interest

The authors declare no conflict of interest.

## Supporting information

Supporting Information

## Data Availability

The data that support the findings of this study are available from the corresponding author upon reasonable request.
