# Supplementary material for: Engineering Shewanella oneidensis‐Carbon Felt Biohybrid Electrode Decorated with Bacterial Cellulose Aerogel‐Electropolymerized Anthraquinone to Boost Energy and Chemicals Production
Source: Adv Sci (Weinh). 2024 Aug 19;11(39):2407599. doi: 10.1002/advs.202407599 (PMC11497010; doi:10.1002/advs.202407599)
Supplement: Supplementary file 1 — Supporting Information [file ADVS-11-2407599-s001.docx]

**Supporting Information (SI)**

**Engineering *Shewanella oneidensis*-carbon felt biohybrid electrode decorated with bacterial cellulose aerogel-electropolymerized anthraquinone to boost energy and chemicals production**

Qijing Liu^1^, Wenliang Xu^1^, Qinran Ding^1^, Yan Zhang^1^, Junqi Zhang^1^, Baocai Zhang^1^, Huan Yu^1^, Chao Li^1^, Longhai Dai^2^, Cheng Zhong^3^, Wenyu Lu^1^, ZhanYing Liu^4,*^, Feng Li^1,*^, Hao Song^1,5,*^

^1^ Frontier Science Center for Synthetic Biology (Ministry of Education), Key Laboratory of Systems Bioengineering, and School of Chemical Engineering and Technology, Tianjin University, Tianjin, 300072, China.

^2^ State Key Laboratory of Biocatalysis and Enzyme Engineering, School of Life Sciences, Hubei University, Wuhan, 430062, China.

^3^ State Key Laboratory of Food Nutrition and Safety, key Laboratory of Industrial Fermentation Microbiology, (ministry of education), Tianjin University of Science and Technology, Tianjin 300457, China.

^4^ Center for Energy Conservation and Emission Reduction in Fermentation Industry in Inner Mongolia, Engineering Research Center of Inner Mongolia for Green Manufacturing in Bio-fermentation Industry, and School of Chemical Engineering, Inner Mongolia University of Technology, Hohhot, 010051, Inner Mongolia, China.

^5^ Haihe Laboratory of Sustainable Chemical Transformations, Tianjin, 300192, China

*Corresponding authors: ZY. Liu (hgxylzy2008@imut.edu.cn); F. Li (feng.li@tju.edu.cn); H. Song (hsong@tju.edu.cn)

**
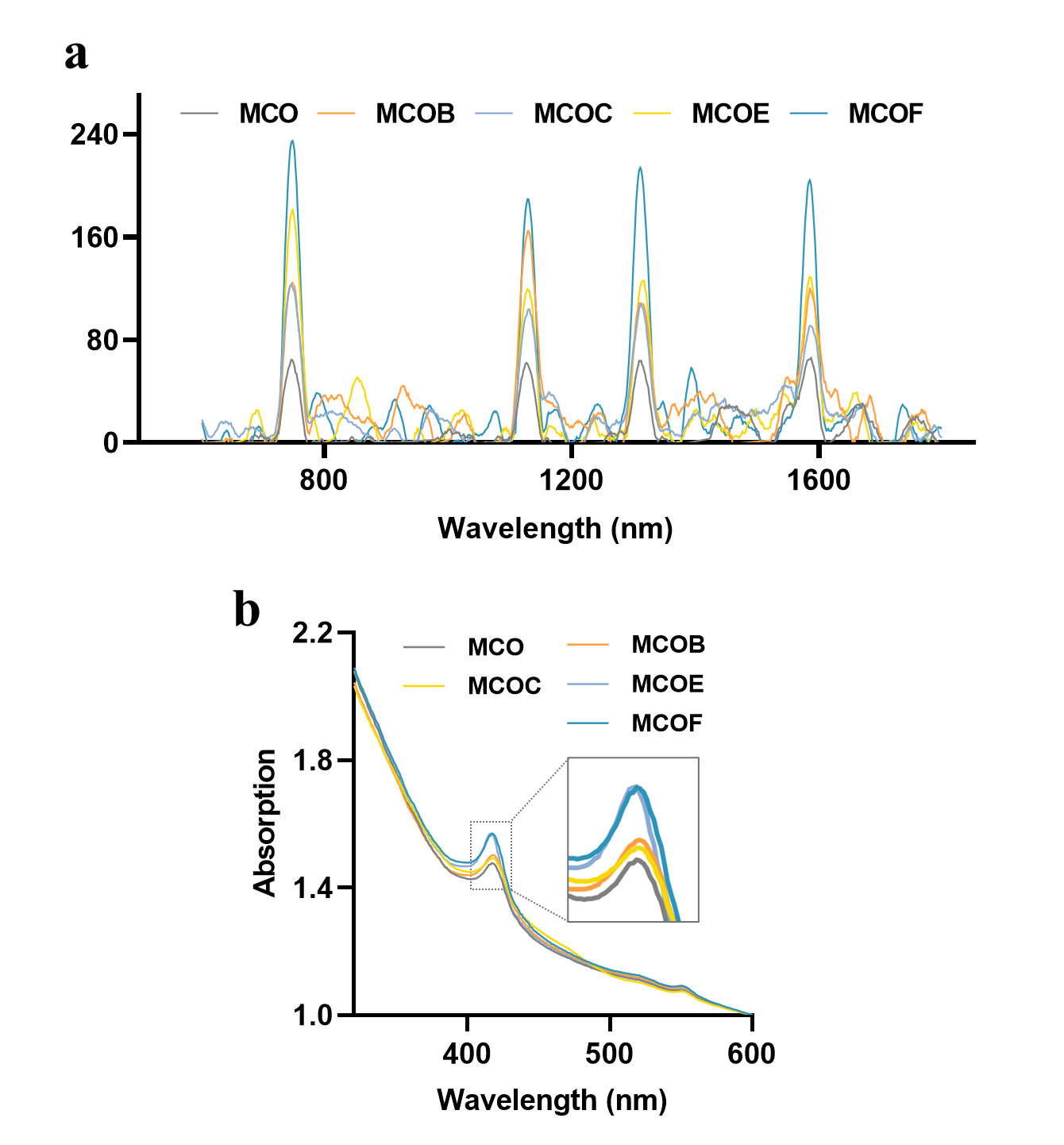
Figure S1. Qualitative characterizations of outer membrane *c*-Cyts of engineered strains. (a)** Raman spectroscopy characterization of engineered strains MCO, MCOB, MCOC, MCOE, and MCOF. **(b)** UV‐visible spectral characterization of *c*-Cyts in the strains MCO, MCOB, MCOC, MCOE, and MCOF.


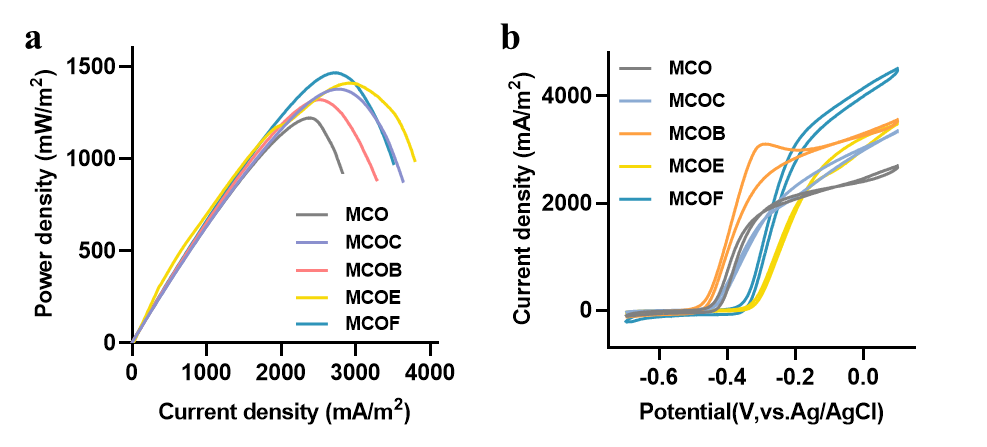


**Figure S2. Electrochemical characterizations of engineered strains MCO, MCOB, MCOC, MCOE, and MCOF in MFCs. (a)** Power densities of strains MCO, MCOB, MCOC, MCOE, and MCOF. **(b)** CV curves of strains MCO, MCOB, MCOC, MCOE, and MCOF.


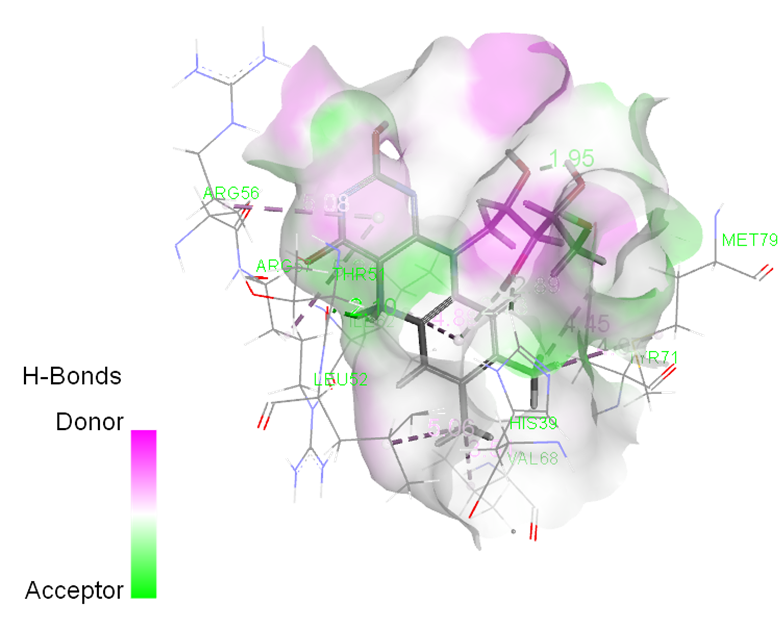


**Figure S3. The display of hydrogen bonding interaction between native *c*-Cyt OmcF and riboflavin** **molecule.** The minimum distance between the native OmcF and flavin molecule was 1.95 Å.


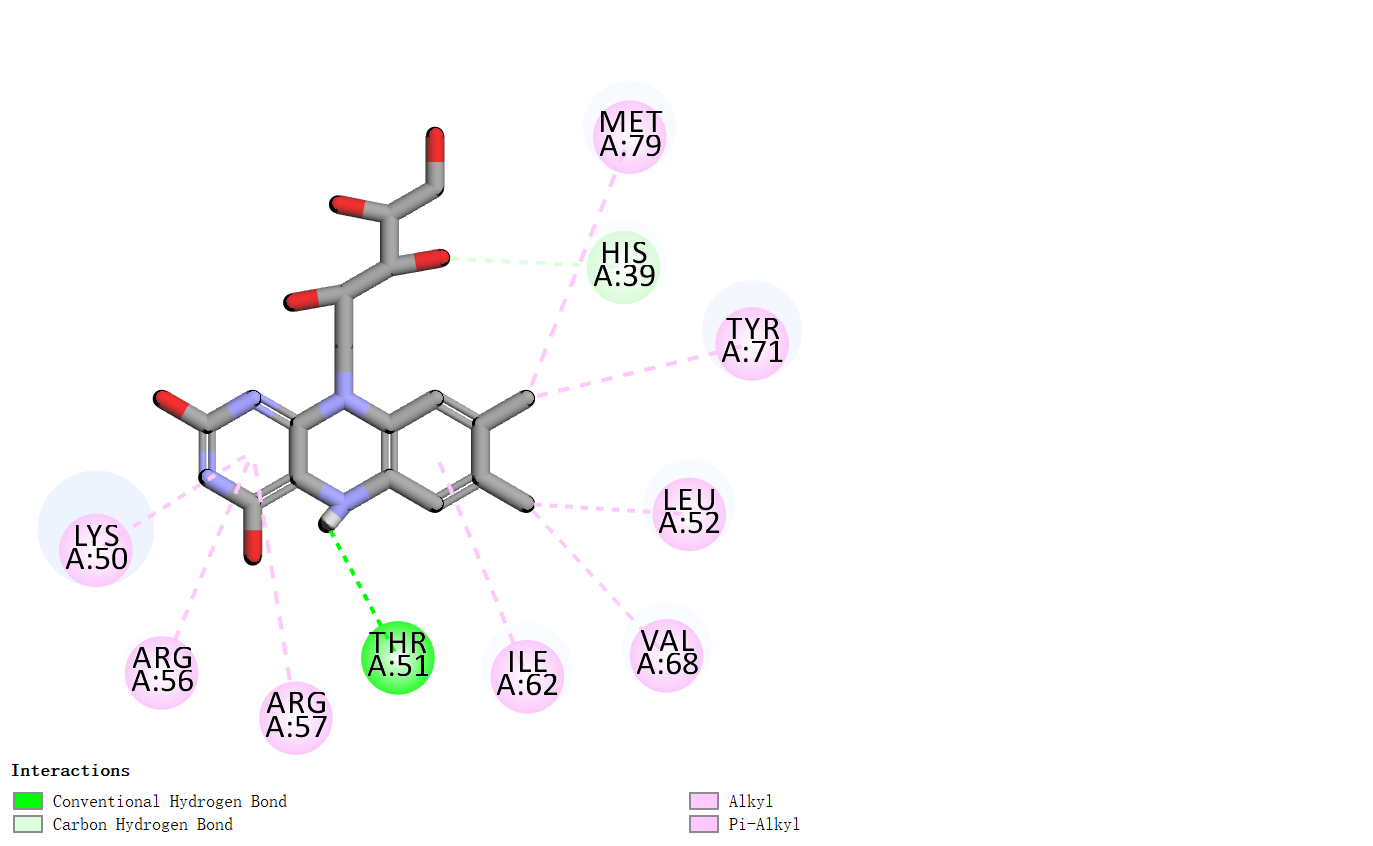


**Figure S4. The 2D display of the interaction between the residues of native OmcF and riboflavin molecule.** The interaction includes the conventional hydrogen bond between T51 and riboflavin molecule, carbon hydrogen bond between H39 and riboflavin molecule, and alkyl between L50/L52/A56/A5 7/I62/V68/ T71/M79 and riboflavin molecule.


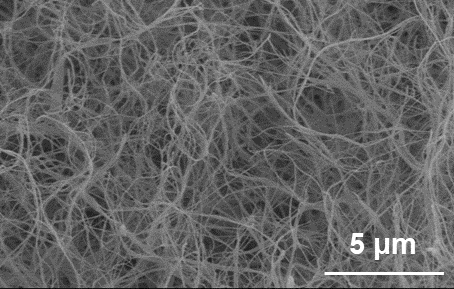


**Figure S5. SEM image of CNF aerogels.** A hierarchically porous carbon nanofiber aerogel (CNFA) prepared from bacterial cellulose (BC) after carbonization.


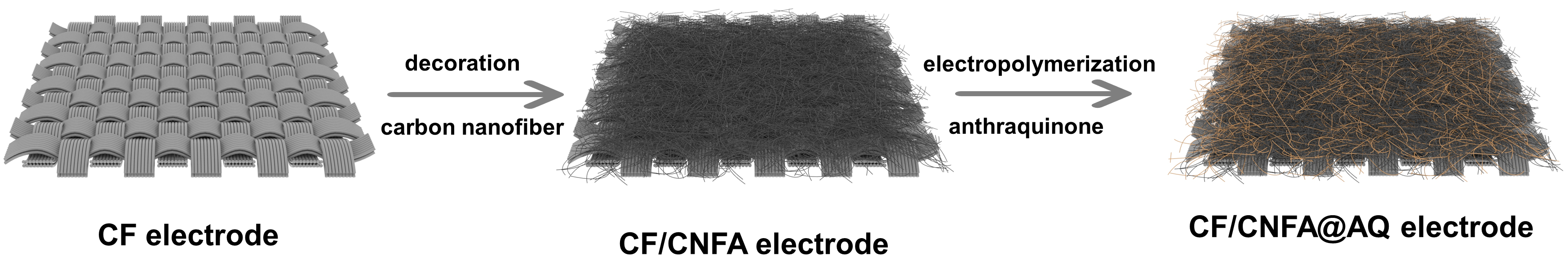


**Figure S6.** **Schematic of decoration process of the CF/CNFA and CF/CNFA@AQ anodes.** To improve cell-electrode interfacial electron transfer, an efficient bacterial cellulose-based aerogel coated anthraquinone anode was constructed. A hierarchically porous carbon nanofiber aerogel (CNFA) was prepared from bacterial cellulose (BC) and further utilized to decorate the carbon felt (CF) anode (named as CF/CNFA anode), followed by the electropolymerization of anthraquinone (AQ) on the surface of aforesaid anode, resulting in the CF/CNFA@AQ anode.


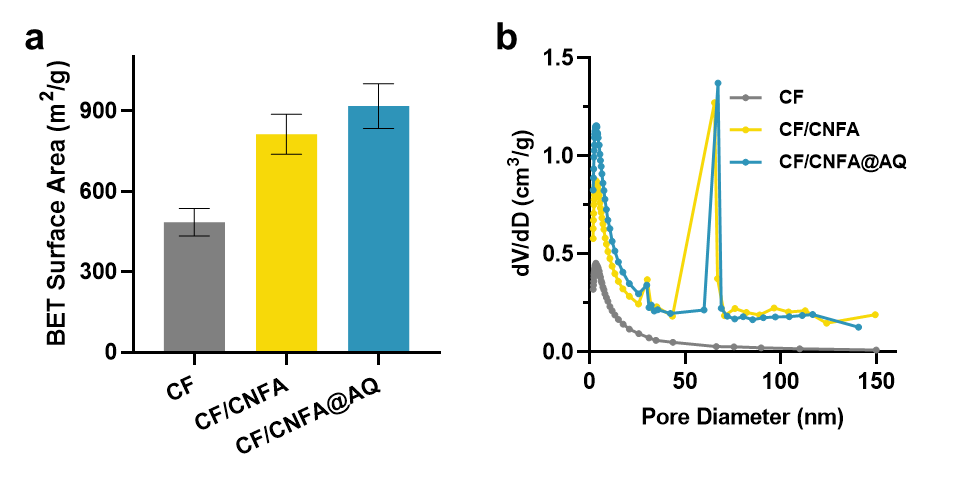


**Figure S7. Surface area and pore size distributions of the CF and decorated anodes.** **(a)** Brunauer–Emmett–Teller **(**BET) surface area of the CF and decorated anodes. **(b)** Barrett–Joyner–Halenda (BJH) pore size distributions of the CF and decorated anodes (CF/CNFA and CF/CNFA@AQ). Data were presented as mean ± SD (n = 3 biological replicates).

**
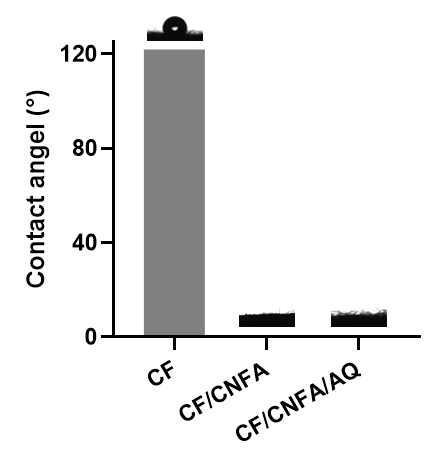
**

**Figure S8. Water contact angle of the CF and decorated anodes (CF/CNFA and CF/CNFA@AQ)**. Data were presented as mean ± SD (n = 3 biological replicates).


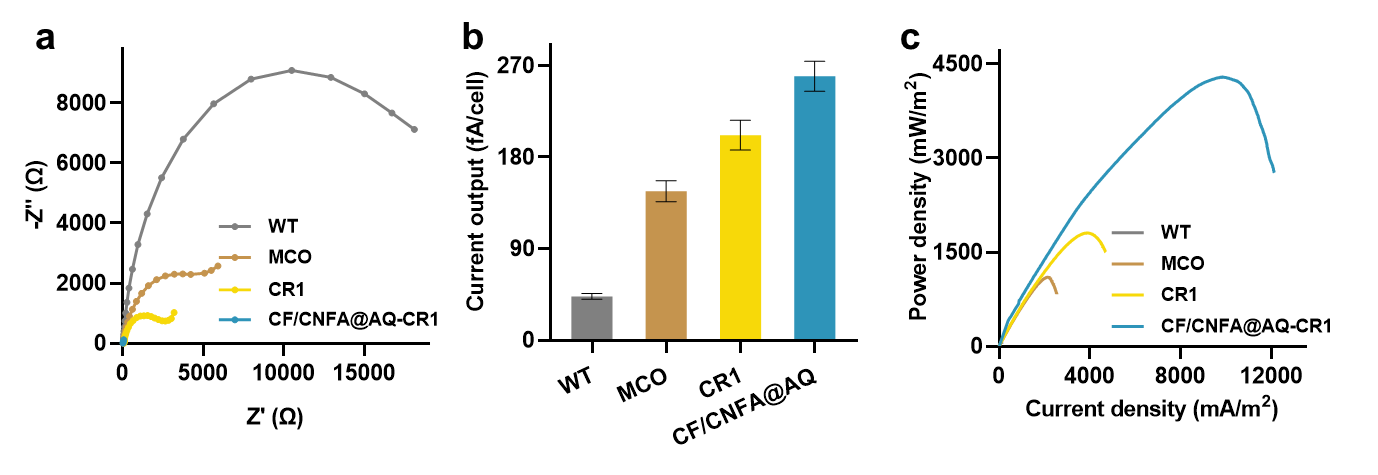


**Figure S9. Boosting cell-electrode interfacial electron transfer rate by constructed modular engineering strategy. (a)** EIS analysis of MC, MCO, CF/CNFA@AQ-CR1, and WT strains, respectively. **(b)** The single-cell current output of MC, MCO, CF/CNFA@AQ-CR1, and WT strains, respectively. **(c)** The output power densities of MC, MCO, CF/CNFA@AQ-CR1, and WT strains, respectively. Data were presented as mean ± SD (n = 3 biological replicates).


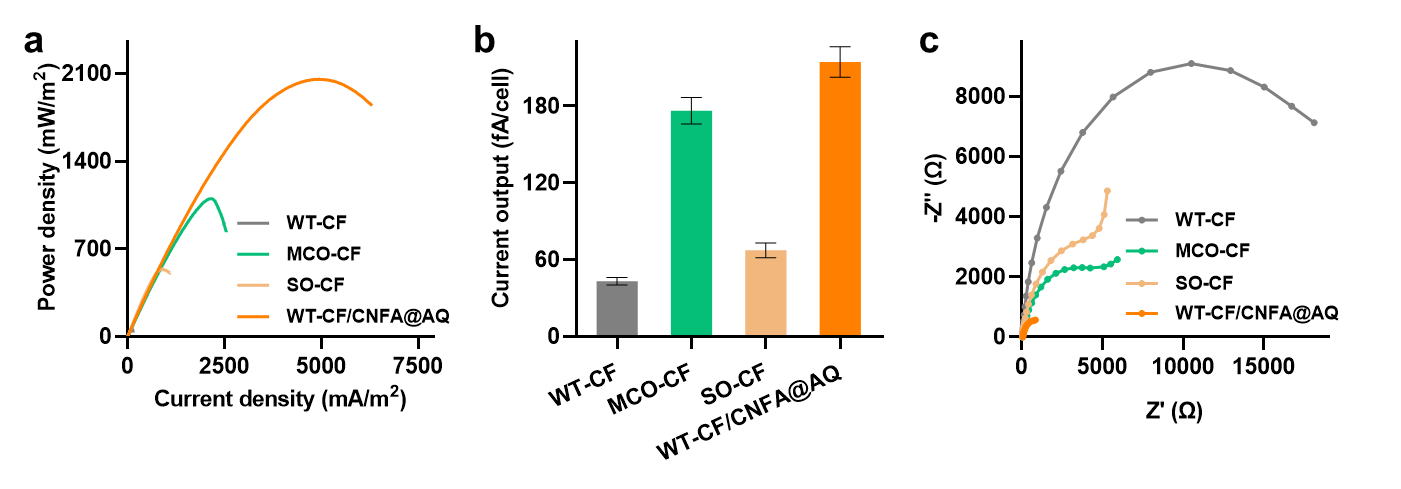


**Figure S10. Boosting cell-electrode interfacial electron transfer rate by individual modular engineering strategy. (a)** The output power densities of MCO-CF, SO-CF and WT- CF/CNFA@AQ, respectively. **(b)** The single-cell current output of MCO-CF, SO-CF and WT- CF/CNFA@AQ, respectively. **(c)** EIS analysis of MCO-CF, SO-CF and WT- CF/CNFA@AQ, respectively. Data were presented as mean ± SD (n = 3 biological replicates).

**Table S1.** Strains used and constructed in this study.

| strains | **Description** | **Source** |
| --- | --- | --- |
| *Shewanella oneidensis* |  |  |
| MR-1 | Lake Oneida isolate | Our Lab |
| WT | MR-1 carrying pYYDT | This study |
| MC | MR-1 carrying pYYDT-*ribADHEC* | This study |
| MCO | MR-1 carrying pYYDT- *ribADHEC-bfe-oprF* | This study |
| MCOB | MR-1 carrying pYYDT- *ribADHEC-bfe-oprF-omcB* | This study |
| MCOC | MR-1 carrying pYYDT- *ribADHEC-bfe-oprF-mtrC* | This study |
| MCOE | MR-1 carrying pYYDT- *ribADHEC-bfe-oprF-omcE* | This study |
| MCOF | MR-1 carrying pYYDT- *ribADHEC-bfe-oprF-omcF* | This study |
| MCOF1 | MR-1 carrying pYYDT- *ribADHEC-bfe-oprF-M1* | This study |
| MCOF2 | MR-1 carrying pYYDT- *ribADHEC-bfe-oprF-M2* | This study |
| MCOF3 | MR-1 carrying pYYDT- *ribADHEC-bfe-oprF-M3* | This study |
| MCOF4 | MR-1 carrying pYYDT- *ribADHEC-bfe-oprF-M4* | This study |
| MCOF5 | MR-1 carrying pYYDT- *ribADHEC-bfe-oprF-M5* | This study |
| MCOF6 | MR-1 carrying pYYDT- *ribADHEC-bfe-oprF-M6* | This study |
| CR1 | MR-1 carrying pYYDT- *ribADHEC-bfe-oprF- BBa_B0032-M5* |  |
| CR2 | MR-1 carrying pYYDT- *ribADHEC-bfe-oprF- BBa_B0030-M5* |  |
| CR3 | MR-1 carrying pYYDT- *ribADHEC-bfe-oprF- BBa_B0033-M5* |  |
| CR4 | MR-1 carrying pYYDT- *ribADHEC-bfe-oprF- BBa_B0035-M5* |  |
| *E. coli* |  |  |
| WM3064 | *ThrB1004 pro thi rpsL hsdS lacZΔM15RP4-1360Δ(araBAD)567 ΔdapA1341::[erm pir]* | Our Lab |

**Table S2.** Plasmids constructed in this study.

| **Plasmids** | **Description** | **Source** |
| --- | --- | --- |
| pYYDT | *pBBR1, kanR, mob, lacI, BBa_B0034* | Our Lab |
| pYYDT-*ribADEHC* | *pBBR1, kanR, mob, lacI, BBa_B0034, Ptac-ribADEHC* | This study |
| pYYDT- *ribADHEC-bfe-oprF* | *pBBR1, kanR, mob, lacI, BBa_B0034, Ptac-ribADEHC-bfe-oprF* | This study |
| pYYDT- *ribADHEC-bfe-oprF-omcB* | *pBBR1, kanR, mob, lacI, BBa_B0034, Ptac-ribADHEC-bfe-oprF-omcB* | This study |
| pYYDT- *ribADHEC-bfe-oprF-mtrC* | *pBBR1, kanR, mob, lacI, BBa_B0034, Ptac-ribADHEC-bfe-oprF-mtrC* | This study |
| pYYDT- *ribADHEC-bfe-oprF-omcE* | *pBBR1, kanR, mob, lacI, BBa_B0034, Ptac-ribADHEC-bfe-oprF-omcE* | This study |
| pYYDT- *ribADHEC-bfe-oprF-omcF* | *pBBR1, kanR, mob, lacI, BBa_B0034, Ptac-ribADHEC-bfe-oprF-omcF* | This study |
| pYYDT- *ribADHEC-bfe-oprF-M1* | *pBBR1, kanR, mob, lacI, BBa_B0034, Ptac-ribADHEC-bfe-oprF-M1* | This study |
| pYYDT- *ribADHEC-bfe-oprF-M2* | *pBBR1, kanR, mob, lacI, BBa_B0034, Ptac-ribADHEC-bfe-oprF-M2* | This study |
| pYYDT- *ribADHEC-bfe-oprF-M3* | *pBBR1, kanR, mob, lacI, BBa_B0034, Ptac-ribADHEC-bfe-oprF-M3* | This study |
| pYYDT- *ribADHEC-bfe-oprF-M4* | *pBBR1, kanR, mob, lacI, BBa_B0034, Ptac-ribADHEC-bfe-oprF-M4* | This study |
| pYYDT- *ribADHEC-bfe-oprF-M5* | *pBBR1, kanR, mob, lacI, BBa_B0034, Ptac-ribADHEC-bfe-oprF-M5* | This study |
| pYYDT- *ribADHEC-bfe-oprF-M6* | *pBBR1, kanR, mob, lacI, BBa_B0034, Ptac-ribADHEC-bfe-oprF-M6* | This study |
| pYYDT- *ribADHEC-bfe-oprF- BBa_B0032-M5* | *pBBR1, kanR, mob, lacI, Ptac- BBa_B0034-ribADHEC-bfe-oprF-BBa_B0032-M5* | This study |
| pYYDT- *ribADHEC-bfe-oprF- BBa_B0032-M5* | *pBBR1, kanR, mob, lacI, Ptac- BBa_B0034-ribADHEC-bfe-oprF-BBa_B0030-M5* | This study |
| pYYDT- *ribADHEC-bfe-oprF- BBa_B0032-M5* | *pBBR1, kanR, mob, lacI, Ptac- BBa_B0034-ribADHEC-bfe-oprF-BBa_B0033-M5* | This study |
| pYYDT- *ribADHEC-bfe-oprF- BBa_B0032-M5* | *pBBR1, kanR, mob, lacI, Ptac- BBa_B0034-ribADHEC-bfe-oprF-BBa_B0035-M5* | This study |

**Table S3.** The sequence of genes used in this study.

| **Genes** | **Sequence** |
| --- | --- |
| *ribA* | atgttccacccaatcgaagaagctttagatgctttaaaaaaaggtgaagttatcatcgttgttgatgatgaagatcgtgaaaacgaaggtgatttcgttgctttagctgaacacgctactccagaagttatcaacttcatggctactcacggtcgtggtttaatctgtactccattatctgaagaaatcgctgatcgtttagatttacacccaatggttgaacacaacactgattctcaccacactgctttcactgtttctatcgatcaccgtgaaactaaaactggtatctctgctcaagaacgttctttcactgttcaagctttattagattctaaatctgttccatctgatttccaacgtccaggtcacatcttcccattaatcgctaaaaaaggtggtgttttaaaacgtgctggtcacactgaagctgctgttgatttagctgaagcttgtggttctccaggtgctggtgttatctgtgaaatcatgaacgaagatggtactatggctcgtgttccagaattaatcgaaatcgctaaaaaacaccaattaaaaatgatcactatcaaagatttaatccaataccgttacaacttaactactttagttgaacgtgaagttgatatcactttaccaactgatttcggtactttcaaagtttacggttacactaacgaagttgatggtaaagaacacgttgctttcgttatgggtgatgttccattcggtgaagaaccagttttagttcgtgttcactctgaatgtttaactggtgatgttttcggttctcaccgttgtgattgtggtccacaattacacgctgctttaaaccaaatcgctgctgaaggtcgtggtgttttattatacttacgtcaagaaggtcgtggtatcggtttaatcaacaaattaaaagcttacaaattacaagaacaaggttacgatactgttgaagctaacgaagctttaggtttcttaccagatttacgtaactacggtatcggtgctcaaatcttacgtgatttaggtgttcgtaacatgaaattattaactaacaacccacgtaaaatcgctggtttagaaggttacggtttatctatctctgaacgtgttccattacaaatggaagctaaagaacacaacaaaaaatacttacaaactaaaatgaacaaattaggtcacttattacacttctaa |
| *ribD* | atggaagaatactacatgaaattagctttagatttagctaaacaaggtgaaggtcaaactgaatctaacccattagttggtgctgttgttgttaaagatggtcaaatcgttggtatgggtgctcacttaaaatacggtgaagctcacgctgaagttcacgctatccacatggctggtgctcacgctgaaggtgctgatatctacgttactttagaaccatgttctcactacggtaaaactccaccatgtgctgaattaatcatcaactctggtatcaaacgtgttttcgttgctatgcgtgatccaaacccattagttgctggtcgtggtatctctatgatgaaagaagctggtatcgaagttcgtgaaggtatcttagctgatcaagctgaacgtttaaacgaaaaattcttacacttcatgcgtactggtttaccatacgttactttaaaagctgctgcttctttagatggtaaaatcgctacttctactggtgattctaaatggatcacttctgaagctgctcgtcaagatgctcaacaataccgtaaaactcaccaatctatcttagttggtgttggtactgttaaagctgataacccatctttaacttgtcgtttaccaaacgttactaaacaaccagttcgtgttatcttagatactgttttatctatcccagaagatgctaaagttatctgtgatcaaatcgctccaacttggatcttcactactgctcgtgctgatgaagaaaaaaaaaaacgtttatctgctttcggtgttaacatcttcactttagaaactgaacgtatccaaatcccagatgttttaaaaatcttagctgaagaaggtatcatgtctgtttacgttgaaggtggttctgctgttcacggttctttcgttaaagaaggttgtttccaagaaatcatcttctacttcgctccaaaattaatcggtggtactcacgctccatctttaatctctggtgaaggtttccaatctatgaaagatgttccattattacaattcactgatatcactcaaatcggtcgtgatatcaaattaactgctaaaccaactaaagaataa |
| *ribH* | atgaacatcatccaaggtaacttagttggtactggtttaaaaatcggtatcgttgttggtcgtttcaacgatttcatcacttctaaattattatctggtgctgaagatgctttattacgtcacggtgttgatactaacgatatcgatgttgcttgggttccaggtgctttcgaaatcccattcgctgctaaaaaaatggctgaaactaaaaaatacgatgctatcatcactttaggtactgttatccgtggtgctactactcactacgattacgtttgtaacgaagctgctaaaggtatcgctcaagctgctaacactactggtgttccagttatcttcggtatcgttactactgaaaacatcgaacaagctatcgaacgtgctggtactaaagctggtaacaaaggtgttgattgtgctgtttctgctatcgaaatggctaacttaaaccgttctttcgaataa |
| *ribE* | atgttcactggtatcatcgaagaaactggtactatcgaatctatgaaaaaagctggtcacgctatggctttaactatcaaatgttctaaaatcttagaagatgttcacttaggtgattctatcgctgttaacggtatctgtttaactgttactgatttcactaaaaaccaattcactgttgatgttatgccagaaactgttaaagctacttctttaaacgatttaactaaaggttctaaagttaacttagaacgtgctatggctgctaacggtcgtttcggtggtcacttcgtttctggtcacgttgatggtactgctgaaatcactcgtatcgaagaaaaatctaacgctgtttactacgatttaaaaatggatccatctttaactaaaactttagttttaaaaggttctatcactgttgatggtgtttctttaactatcttcggtttaactgaagatactgttactatctctttaatcccacacactatctctgaaactatcttctctgaaaaaactatcggttctaaagttaacatcgaatgtgatatgatcggtaaatacatgtaccgtttcttacacaaagctaacgaaaacaaaactcaacaaactatcactaaagctttcttatctgaaaacggtttctaa |
| *ribC* | gtgaaaactatccacatcactcacccacaccacttaatcaaagaagaacaagctaaatctgttatggctttaggttacttcgatggtgttcacttaggtcaccaaaaagttatcggtactgctaaacaaatcgctgaagaaaaaggtttaactttagctgttatgactttccacccacacccatctcacgttttaggtcgtgataaagaaccaaaagatttaatcactccattagaagataaaatcaaccaaatcgaacaattaggtactgaagttttatacgttgttaaattcaacgaagttttcgcttctttatctccaaaacaattcatcgatcaatacatcatcggtttaaacgttcaacacgctgttgctggtttcgatttcacttacggtaaatacggtaaaggtactatgaaaactatgccagatgatttagatggtaaagctggttgtactatggttgaaaaattaactgaacaagataaaaaaatctcttcttcttacatccgtactgctttacaaaacggtgatgttgaattagctaacgttttattaggtcaaccatacttcatcaaaggtatcgttatccacggtgataaacgtggtcgtactatcggtttcccaactgctaacgttggtttaaacaactcttacatcgttccaccaactggtgtttacgctgttaaagctgaagttaacggtgaagtttacaacggtgtttgtaacatcggttacaaaccaactttctacgaaaaacgtccagaacaaccatctatcgaagttaacttattcgatttcaaccaagaagtttacggtgctgctatcaaaatcgaatggtacaaacgtatccgttctgaacgtaaattcaacggtatcaaagaattaactgaacaaatcgaaaaagataaacaagaagctatccgttacttctctaacttacgtaaataa |
| *omcB* | atgttcacaagaaagattcaaaaaacagcactagccatgctgatctctggcgcaatggcaggcacagcctatgccgctccagaagtactagccgattttcacggtgaaatgggtggctgcgatagctgccacgtatcagacaaaggtggcgtgactaacgacaacctgacccatgagaatggccaatgtgttagctgccacggtgacttaaaagaactggctgcagcagcgcctaaagataaagtttctccgcacaaatctcacttaattggtgaaatcgcttgtacgagctgccacaaaggccacgaaaaatcagtagcttattgtgatgcttgccatagcttcggcttcgatatgccatttggtggcaagtgggaacgtaagtttgtacctgttgatgcagacaaagcagcacaagataaagccattgctgctggtgtgaaagaaaccacagacgttgtaattatcggctctggtggtgctggtcttgccgctgccgtatctgcccgtgatgctggcgcgaaagtgattctgttagaaaaagaacctatcccaggtggtaacactaaactggctgccggtggtatgaacgccgcagaaactaagccacaggctaagttaggtatcgaagataagaaacaaatcatgatcgacgacactatgaaaggtggccgcaacatcaacgatcctgaattagttaaagtactggctaacaactcttcagactcaatcgattggttaacctctatgggtgccgacatgactgacgtgggtcgtatgggtggcgcgagcgttaaccgtagtcaccgtccaaccggtggtgcaggtgttggtgctcacgtagcacaagtgctgtgggacaacgccgttaagcgtggtactgatattcgcttaaacagccgcgttgtgcgcatccttgaagatgcaagcggtaaagtgaccggcgttttagtgaaaggtgaatacacaggttactatgtgatcaaagccgatgcagtagtcattgcagcaggtggttttgcgaaaaacaacgaacgtgtttctaaatacgatcctaagttaaaaggctttaaagcgaccaaccacccaggtgcgacgggtgacggcttagacgtagctctacaagcgggcgcagcaacacgtgacttagaatacatccaagctcacccaacttactctccagcgggtggcgtgatgatcaccgaggcagtacgtggtaacggtgcaatcgtggtgaaccgtgaaggtaatcgttttatgaacgaaatcaccacccgtgataaagcgtctgcggcgattctgcaacaaaaaggtgaaagcgcttacctagtattcgatgactctatccgtaagagcttgaaggccatcgaaggttatgttcacctgaacattgtaaaagaaggtaaaaccatcgaagagttagcgaaacaaatcgatgtacctgcagctgaattggcaaaaacagtaacagcctacaacggtttcgttaaatcaggtaaagatgctcaatttgaacgtcctgatttaccacgtgaattagtggtagctcctttctacgccttagaaattgcaccagcggttcaccacactatgggtggtctggtgattgatactaaagccgaagtgaagagtgagaaaaccggtaaacctatcactggtttatacgctgcaggcgaagtgactggtggtgttcacggtgctaaccgtttaggtggtaacgctatctctgatatcgtcacctacggccgtatcgcgggtgcatctgccgctaaattcgctaaagataat |
| *mtrC* | atgatgaacgcacaaaaatcaaaaatcgcactgctgctcgcagcaagtgccgtcacaatggccttaaccggctgtggtggaagcgatggtaataacggcaatgatggtagtgatggtggtgagccagcaggtagcatccagacgttaaacctagatatcactaaagtaagctatgaaaatggtgcacctatggtcactgttttcgccactaacgaagccgacatgccagtgattggtctcgcaaatttagaaatcaaaaaagcactgcaattaataccggaaggggcgacaggcccaggtaatagcgctaactggcaaggcttaggctcatcaaagagctatgtcgataataaaaacggtagctatacctttaaattcgacgccttcgatagtaataaggtctttaatgctcaattaacgcaacgctttaacgttgtttctgctgcgggtaaattagcagacggaacgaccgttcccgttgccgaaatggttgaagatttcgacggccaaggtaatgcgccgcaatatacaaaaaatatcgttagccacgaagtatgtgcttcttgccacgtagaaggtgaaaagatttatcaccaagctactgaagtcgaaacttgtatttcttgccacactcaagagtttgcggatggtcgcggcaaaccccatgtcgcctttagtcacttaattcacaatgtgcataatgccaacaaagcttggggcaaagacaataaaatccctacagttgcacaaaatattgtccaagataattgccaagtttgtcacgttgaatccgacatgctcaccgaggcaaaaaactggtcacgtattccaacaatggaagtctgttctagctgtcacgtagacatcgattttgctgcgggtaaaggccactctcaacaactcgataactccaactgtatcgcctgccataacagcgactggactgctgagttacacacagccaaaaccaccgcaactaagaacttgattaatcaatacggtatcgagactacctcgacaattaataccgaaactaaagcagccacaattagtgttcaagttgtagatgcgaacggtactgctgttgatctcaagaccatcctgcctaaagtgcaacgcttagagatcatcaccaacgttggtcctaataatgcaaccttaggttatagtggcaaagattcaatatttgcaatcaaaaatggagctcttgatccaaaagctactatcaatgatgctggcaaactggtttataccactactaaagacctcaaacttggccaaaacggcgcagacagcgacacagcatttagctttgtaggttggtcaatgtgttctagcgaaggtaagtttgtagactgtgcagaccctgcatttgatggtgttgatgtaactaagtataccggcatgaaagcggatttagcctttgctactttgtcaggtaaagcaccaagtactcgccacgttgattctgttaacatgacagcctgtgccaattgccacactgctgagttcgaaattcacaaaggcaaacaacatgcaggctttgtgatgacagagcaactatcacacacccaagatgctaacggtaaagcgattgtaggccttgacgcatgtgtgacttgtcatactcctgatggcacctatagctttgccaaccgtggtgcgctagagctaaaactacacaaaaaacacgttgaagatgcctacggcctcattggtggcaattgtgcctcttgtcactcagacttcaaccttgagtctttcaagaagaaaggcgcattgaatactgccgctgcagcagataaaacaggtctatattctacgccgatcactgcaacttgtactacctgtcacacagttggcagccagtacatggtccatacgaaagaaaccctggagtctttcggtgcagttgttgatggcacaaaagatgatgctaccagtgcggcacagtcagaaacctgtttctactgccataccccaacagttgcagatcacactaaagtgaaaatgtaa |
| *omcE* | gtggaggcactgaccatgcgtatcggcgaactgtcccgccacagtggctgcgacatcgaaaccatccgctattacgaacgtgaagggctgctggacgcaccgcagcgcgaggacaacggctatcgccgctatggcgacgggcacctcgtccagctcaacttcgtgcgccactgccgctcgctgggcatgagcctggccgacgtgcgcaagctgcgcgacttccagcgcaatccgtcactcgcgtgcgacgacatcaatacgctgttggaccgccagatcgagcagatccacgcccagcgtgtttcgctggaagcgctggaaggtcagttgcgcacgctgcgccatacctgcgagaacccaaatccgcatccggccagcgagtgcggcatcctgcagaacctgcagcaggcagccgagggcgcggcctgcgagtgccacccacgacactag |
| *omcF* | atgcgtggtttagctccagttgctgcttgtatggctttagctttagctgctggttgttctggtggttctggtgctggtggtggtgaattattcgctactcactgtgctggttgtcacccacaaggtggtaacactgttcacccagaaaaaactttagctcgtgctcgtcgtgaagctaacggtatccgtactgttcgtgatgttgctgcttacatccgtaacccaggtccaggtatgccagctttcggtgaagctatgatcccaccagctgatgctttaaaaatcggtgaatacgttgttgcttctttcccataa |
| *M1* | MRGLAPVAACMALALAAGCSGGSGAGGEELFATHCAGCHPDGGNTVHPEKTLARARREANGIRTVRDVAAYIRNPGPGMPAFDEAMIPPADALKIGEYVVASFP* |
| *M2* | MRGLAPVAACMALALAAGCSGGSGAGGEELFKTHCAGCHPDGGNTVNPEKTLHRADREANGIRTVRDVAAYIRNPGPGMPAFDEAMIPPADALKIGEYVVASFP* |
| *M3* | MRGLAPVAACMALALAAGCSGGSGAGGEELFKTHCAGCHPDGGNTVNPEKTLHRADREANGIRTVRDVAAYIRNPGPGMPAFDEATIPPADALKIGEYVLASFP* |
| *M4* | MRGLAPVAACMALALAAGCSGGSGAGGEELFKTHCAGCHPDGGNTVNPEKTLHRADREANGIRTVRDVAAYMRNPGPGMPAFDEKTIPPKDALKIGEYVLKSFP* |
| *M5* | MRGLAPVAACMALALAAGCSGGSGAGGEELFKTHCAGCHPDGGNTVNPEKTLHRADREANGIRTVRDVAAYMRNPGPGMPAFDEKTIPDKDAKKIGEYVLKSFP* |
| *M6* | MRGLAPVAACMALALAAGCSGGSGAGGEELFKTHCAGCHPDGGNTINPEKTLHRKDREANGIRTVRDIAKYMRNPGPGMPAFDEKTIPDKDAKKIGEYVLKSFP* |
| *BBa_B0030* | attaaagaggagaaa |
| *BBa_B0032* | tcacacaggaaag |
| *BBa_B0033* | tcacacaggac |
| *BBa_B0034* | aaagaggagaaa |
| *BBa_B0035* | attaaagaggagaa |

**Table S5.** Summary of the reported performance of the biohybrid electrodes constructed by coupling genetic engineering exoelectrogens and electrode decoration.

| NO. | Exoelectrogens | Anode/ Cathode/ Catholyte | Engineering strategy description | Power density(W/m^2^) | | Ref. |
| --- | --- | --- | --- | --- | --- | --- |
| 1 | *S. oneidensis* MR-1 | Graphite felt/ Graphite felt/ O_2_ | Discruption of the putative cell surface polysaccharide biosynthesis gene *SO3177* | 0.065 | 1 | |
| 2 | *S. oneidensis* MR-1 | NM | Disrupting putrescine biosynthesis gene *speF* for enhancing capability in biofilm formation under both static and hydrodynamic conditions. | NM | 2 | |
| 3 | *E. coli*-*Bacillus subtilis* -*S. oneidensis* three-species microbial consortium | Carbon cloth/ Carbon cloth/ Ferricyanide | *E. coli* was designed to fermenter via knocking out of the *pflB* gene, which digested glucose to produce lactate as carbon source and electron donor; *B. subtilis* overproduced riboflavin with overexpressing the *ribABDEC* cluster and reducing glycolysis and ED pathways; and *S. oneidensis* served as the exoelectrogen to generate electricity. | 0.241 | 3 | |
| 4 | *S. oneidensis* MR-1 | Carbon cloth/ Carbon cloth/ Ferricyanide | Overexpressing the NDH II enzyme *ndh* II (Gene ID: 4921489 from *S. loihica* PV-4 in *S. oneidensis* MR-1 strain for enhancing electron trans-IM movement. | 0.37 | 4 | |
| 5 | *G. sulfurreducens* | Graphite rod/ Graphite cloth/ Ferricyanide | Discruption of the *GSU1240* for biofilm formation more effectively. | ~1.3 | 5 | |
| 6 | Fungus (*S. cerevisiae*)-bacteria (*S. oneidensis*) microbial consortium | Carbon cloth/ Carbon cloth/ Ferricyanide | The fermenter, *S. cerevisiae*, was knocked out ethanol pathway and incorporated lactic acid pathway for promoted lactic acid production from glucose. The exoelectrogen, *S. oneidensis*, was enhanced in membrane permeability with expressing *oprF*. | 0.123 | 6 | |
| 7 | Microbial consortium in methane-consuming sludge | Carbon fibre brush/ Carbon fibre brush/ Ferricyanide | Engineering *M. acetivorans* to overexpress methyl-coenzyme M reductase (Mcr) for convert methane to acetate, which serve as electron donor to *G. sulfurreducens* for electrical current generation. | 0.168 | 7 | |
| 8 | *E. coli* | Carbon cloth/ Carbon cloth/ Ferricyanide | Disruption of *arcA* for enhancing activation of the citric acid cycle for glycerol oxidation. | 0.116 | 8 | |
| 9 | *S. oneidensis* MR-1 | Graphite felt/ Graphite felt/ O_2_ | Screening mutants *SO1860* from a random transposon insertion library for altered colony morphology | 0.087 | 9 | |
| 10 | *P. aeruginosa PAO1* | Carbon cloth/ Carbon cloth/ Ferricyanide | Overexpression of *rhlA* for rhamnolipids synthesis, which enhanced electron shuttle production and increased bacteria attachment on the anode | 0.0094 | 10 | |
| 11 | P. aeruginosa PAO1 | Garbon cloth/ Pt-loaded arbon cloth/O_2_ | Overexpression of phzM for enhancing PYO synthsis in P. aeruginosa | 1.668 | 11 | |
| 12 | *S. oneidensis* MR-1 | Stainless steel Carbon cloth/ Ferricyanide | Hybridized curli nanofibers fused with a metal- binding domain were heterogeneously expressed onto the cell surface, which realized efficient cell binding with the SS electrode. | 0.055 | 12 | |
| 13 | *Clostridium ljungdahlii* | Carbon felt/ Carbon felt/ Ferricyanide | Overexpression of the *Fdh* gene enabled to increase NADH regeneration and alter the NADH/NAD+ ratio. | 0.035 | 13 | |
| 14 | *S. oneidensis* MR-1 | Carbon cloth/ Carbon cloth/ Ferricyanide | Enhancing flavins biosynthesis and transportation via regulating RibADEHC and oprF expression with promoter and ribosome binding site (RBS) engineering in a hydrophobic chassis. | 1.12 | 14 | |
| 15 | *S. oneidensis* MR-1 | Carbon cloth/ Carbon cloth/ Ferricyanide | Heterologously expressing RibADEHC originating from *B. subitlis* for enhancing flavin synthesis. | 0.233 | 15 | |
| 16 | *P. aeruginosa* PAO1 | Carbon felt/ Carbon felt/O_2_ | Overexpressing the *PqsE* effector in a PQS negative ∆*pqsC* mutant for producing higher concentrations of phenazines under anaerobic conditions | ~0.0003 | 16 | |
| 17 | *S. oneidensis* MR-1 | Carbon cloth/ Carbon cloth/ Ferricyanide | Engineering and driving the metabolic flux toward the enhancement of intracellular NADH regeneration with modular metabolic engineering strategy. | 0.106 | 17 | |
| 18 | *P. aeruginosa* | Carbon cloth/ Pt-loaded/O2 | Overexpression of NAD synthetase *nadE* | 0.4 | 18 | |
| 19 | *E. coli* | Carbon cloth/ Carbon cloth/ Ferricyanide | Heterologously expressing a porin protein OprF in *E. coli*, for increasing membrane permeability and bioelectricity generation. | NM | 19 | |
| 20 | *S. oneidensis* MR-1 | Carbon cloth/ Carbon cloth/ Ferricyanide | Assembling one of the xylose transporters with one of intracellular xylose metabolic pathways, to enable *S. oneidensis* to directly utilize xylose as the sole carbon source for bioelectricity production. | 0.002 | 20 | |
| 21 | *S. oneidensis* MR-1 | Carbon felt/ Carbon felt/ Ferricyanide | Expression of *mtrC-mtrA-mtrB* and *ribD-ribC-ribBA-ribE* | 0.037 | 21 | |
| 22 | *E. coli* -*S. oneidensis* MR-1 microbial consortium | Carbon cloth/ Carbon cloth/ Ferricyanide | Riboflavin synthetic pathway RibADEHC was incorporated into fermenter, *E. coli*, for facilitating flavins production, and highly hydrophobic *S. oneidensis* was adopted as the exoelectrogen to increase its adhesion to the carbon electrode. | 0.728 | 22 | |
| 23 | *P.aeruginosa* PAO1 | Carbon cloth/ Carbon cloth/ Ferricyanide | Knocking out global regulator (RpoS) of *P. aeruginosa* for enhancing biofilm formation and adaption EET to the redox species. | NM | 23 | |
| 24 | *K. pneumoniae*-*S. oneidensis* microbial consortium | Carbon cloth/ Carbon cloth/ Ferricyanide | Disrupting phosphotransacetylase Pta as well as alcohol dehydrogenase AdhE and expressing lactate dehydrogenase LdhD in *K. pneumoniae* for converting glucose and xylose into lactate to feed *S. oneidensis*. Flavins biosynthetic pathway was expressed in a highly hydrophobic *speF*-deletion *S. oneidensis* mutant for enhancing EET using corn straw hydrolyzates as electron donor. | 0.0235 | 24 | |
| 25 | *S. oneidensis* MR-1 | Graphite felt/ Graphite felt electroplated with a platinum/O_2_ | Introduction the glucose facilitator *glf* and glucokinase *glk* from *Z. mobilis* into *S. oneidensis* MR-1 for electricity generation using glucose as a sole carbon and electron source under anaerobic condition. | NM | 25 | |
| 26 | *S. oneidensis* MR-1 | Graphite felt/ Graphite felt/ O_2_ | Gene-konckout mutants of *SO3350* that adapt to an electrode-respiring | ~0.11 | 26 | |
| 27 | *S. oneidensis* MR-1 | CF/CNFA@AQ  Ferricyanide | heterologous riboflavin synthesis and secretion pathway was constructed; outer membrane *c*-Cyts OmcF was screened and optimized via protein engineering; a *S. oneidensis*-carbon felt biohybrid electrode decorated with bacterial cellulose aerogel and electropolymerized anthraquinone was constructed to boost the interfacial electron transfer | 4.29 ± 0.2 | This study | |

Reference

1. Kouzuma A., Meng X. Y., Kimura N., Hashimoto K., Watanabe K. Disruption of the putative cell surface polysaccharide biosynthesis gene *SO3177* in *Shewanella oneidensis* MR-1 enhances adhesion to electrodes and current generation in microbial fuel cells. *Appl. Environ. Microbiol.* 2010, *76* (13), 4151-7.

2. Ding Y., Peng N., Du Y., Ji L., Cao B. Disruption of putrescine biosynthesis in *Shewanella oneidensis* enhances biofilm cohesiveness and performance in Cr(VI) immobilization. *Appl. Environ. Microbiol.* 2014, *80* (4), 1498-506.

3. Liu Y., Ding M., Ling W., Yang Y., Zhou X., Li B., Chen T., Nie Y., Wang M., Zeng B., Li X., Liu H., Sun B., Xu H., Zhang J., Jiao Y., Hou Y., Yang H., Xiao S., Lin Q., He X., Liao W., Jin Z., Xie Y., Zhang B., Li T., Lu X., Li J., Zhang F., Wu X., Song H., Yuan Y. A three-species microbial consortium for power generation. *Energy Environ. Sci.* 2017, *10* (7), 1600-1609.

4. Tao L., Xie M., Chiew G. G., Wang Z., Chen W. N., Wang X. Improving electron trans-inner membrane movements in microbial electrocatalysts. *Chem. Commun.* 2016, *52* (37), 6292-5.

5. Leang C., Malvankar N. S., Franks A. E., Nevin K. P., Lovley D. R. Engineering *Geobacter sulfurreducens* to produce a highly cohesive conductive matrix with enhanced capacity for current production. *Energy Environ. Sci.* 2013, *6* (6).

6. Lin T., Bai X., Hu Y., Li B., Yuan Y.-J., Song H., Yang Y., Wang J. Synthetic *Saccharomyces cerevisiae*-*Shewanella oneidensis* consortium enables glucose-fed high-performance microbial fuel cell. *AIChE J.* 2017, *63* (6), 1830-1838.

7. McAnulty M. J., Poosarla V. G., Kim K. Y., Jasso-Chavez R., Logan B. E., WoodT. K. Electricity from methane by reversing methanogenesis. *Nat. Commun.* 2017, *8*, 15419.

8. Liu J., Yong Y.-C., Song H., Li C. M. Activation enhancement of citric acid cycle to promote bioelectrocatalytic activity of arcA knockout *Escherichia coli* toward high-performance microbial fuel cell. *ACS Catal.* 2012, *2* (8), 1749-1752.

9. Kouzuma1 A., Oba H., Tajima N., Hashimoto K., Watanabe K. Electrochemical selection and characterization of a high current-generating *Shewanella oneidensis* mutant with altered cell-surface morphology and biofilm-related gene expression. *BMC Microbiol.* 2014, 14, 190-211.

10. Zheng T., Xu Y. S., Yong X. Y., Li B., Yin D., Cheng Q. W., Yuan H. R.,Yong Y. C. Endogenously enhanced biosurfactant production promotes electricity generation from microbial fuel cells. *Bioresour. Technol.* 2015, *197*, 416-21.

11. Yong X. Y., Shi D. Y., Chen Y. L., Feng J., Xu, L., Zhou J., Wang S. Y., Yong Y. C., Sun Y. M., OuYang P. K., Zheng T. Enhancement of bioelectricity generation by manipulation of the electron shuttles synthesis pathway in microbial fuel cells. *Bioresour. Technol.* 2014, *152*, 220-4.

12. Suo D., Fang Z., Yu Y. Y., Yong Y. C. Synthetic curli enables efficient microbial electrocatalysis with stainless‐steel electrode. *AIChE J.* 2019, *66* (4).

13. Han S., Gao X.-y., Ying, H.-j., Zhou C. C. NADH gene manipulation for advancing bioelectricity in Clostridium ljungdahlii microbial fuel cells. *Green Chem.* 2016, *18* (8), 2473-2478.

14. Lin T., Ding W., Sun L., Wang L., Liu C.-G., Song H. Engineered *Shewanella oneidensis* -reduced graphene oxide biohybrid with enhanced biosynthesis and transport of flavins enabled a highest bioelectricity output in microbial fuel cells. *Nano Energy* 2018, *50*, 639-648.

15. Yang Y., Ding Y., Hu Y., Cao B., Rice S. A., Kjelleberg S., Song H. Enhancing bidirectional electron transfer of *Shewanella oneidensis* by a synthetic flavin pathway. *ACS Synth. Biol.* 2015, *4* (7), 815-23.

16. Wang K., Bhandari V., Chepustanova S., Huber G., O’Hara S., O’Hern C. S., Shattuck M. D., Kirby M. Which biomarkers reveal neonatal sepsis? *PloS One* 2013, *8* (12), e82700.

17. Li F., Li Y., Sun L., Chen X., An X., Yin C., Cao Y., Wu H., Song H. Modular engineering intracellular NADH regeneration boosts extracellular electron transfer of *Shewanella oneidensis* MR-1. *ACS Synth. Biol.* 2018, *7* (3), 885-895.

18. Yong X. Y., Feng J., Chen Y. L., Shi D. Y., Xu Y. S., Zhou J., Wang S. Y., Xu L., Yong Y. C., Sun Y. M., Shi C. L., OuYang P. K., Zheng T. Enhancement of bioelectricity generation by cofactor manipulation in microbial fuel cell. *Biosens. Bioelectron.* 2014, *56*, 19-25.

19. Yong Y. C., Yu Y. Y., Yang Y., Liu J., Wang J. Y., Song H. Enhancement of extracellular electron transfer and bioelectricity output by synthetic porin. *Biotechnol. Bioeng.* 2013, *110* (2), 408-16.

20. Li F., Li Y., Sun L., Li X., Yin C., An X., Chen X., Tian Y., Song H. Engineering *Shewanella oneidensis* enables xylose-fed microbial fuel cell. *Biotechnol. Biofuels* 2017, *10* (1).

21. Min D., Cheng L., Zhang F., Huang X., Li D., Liu D., Lau T., Mu Y., Yu H. Enhancing extracellular electron transfer of *Shewanella oneidensis* MR-1 through coupling improved flavin synthesis and metal-reducing conduit for pollutant degradation. *Environ. Sci. Technol.* 2017, *51*(9), 5082-5089.

22. Yang Y., Wu Y., Hu Y., Cao Y., Poh C. L., Cao B., Song H. Engineering electrode-attached microbial consortia for high-performance vylose-fed microbial fuel cell. *ACS Catal.* 2015, *5* (11), 6937-6945.

23. Yu Y.-Y., Fang Z., Gao L., Song H., Yang L., Mao B., Shi W., Yong Y.-C. Engineering of bacterial electrochemical activity with global regulator manipulation. *Electrochem. Commun.* 2018, *86*, 117-120.

24. Li F., Yin C., Sun L., Li Y., Guo X., Song H. Synthetic *Klebsiella pneumoniae*-*Shewanella oneidensis* consortium enables glycerol-fed high-performance microbial fuel cells. *Biotechnol. J.* 2018, *13* (5).

25. Choi D., Lee S. B., Kim S., Min B., Choi I. G., Chang I. S. Metabolically engineered glucose-utilizing Shewanella strains under anaerobic conditions. *Bioresour. Technol.* 2014, *154*, 59-66.

26. Tajima N., Kouzuma A., Hashimoto K, Watanabe K. Selection of *Shewanella oneidensis* MR-1 gene-knockout mutants that adapt to an electrode-respiring condition. *Biosci. Biotechnol. Biochem.* 2011, *75* (11), 2229-33.
